# Characterization of Catechol-1,2-Dioxygenase (Acdo1p) From *Blastobotrys raffinosifermentans* and Investigation of Its Role in the Catabolism of Aromatic Compounds

**DOI:** 10.3389/fmicb.2022.872298

**Published:** 2022-06-03

**Authors:** Anna Meier, Sebastian Worch, Anja Hartmann, Marek Marzec, Hans-Peter Mock, Rüdiger Bode, Gotthard Kunze, Falko Matthes

**Affiliations:** ^1^Institute of Plant Genetics and Crop Plant Research, Gatersleben, Germany; ^2^Institute of Biology, Biotechnology and Environmental Protection, Faculty of Natural Sciences, University of Silesia, Katowice, Poland; ^3^Institute of Microbiology, University of Greifswald, Greifswald, Germany

**Keywords:** biodegradation, aromatic acids, protocatechuic acid, antimicrobial plant substances, *cis*, cis-muconic acid, *Blastobotrys*

## Abstract

Gallic acid, protocatechuic acid, catechol, and pyrogallol are only a few examples of industrially relevant aromatics. Today much attention is paid to the development of new microbial factories for the environmentally friendly biosynthesis of industrially relevant chemicals with renewable resources or organic pollutants as the starting material. The non–conventional yeast, *Blastobotrys raffinosifermentans*, possesses attractive properties for industrial bio-production processes such as thermo- and osmotolerance. An additional advantage is its broad substrate spectrum, with tannins at the forefront. The present study is dedicated to the characterization of catechol-1,2-dioxygenase (Acdo1p) and the analysis of its function in *B. raffinosifermentans* tannic acid catabolism. Acdo1p is a dimeric protein with higher affinity for catechol (*K*_M_ = 0.004 ± 0.001 mM, *k*_cat_ = 15.6 ± 0.4 s^–1^) than to pyrogallol (*K*_M_ = 0.1 ± 0.02 mM, *k*_cat_ = 10.6 ± 0.4 s^–1^). It is an intradiol dioxygenase and its reaction product with catechol as the substrate is *cis,cis*-muconic acid. *B. raffinosifermentans* G1212/YIC102-AYNI1-ACDO1-6H, which expresses the *ACDO1* gene under the control of the strong nitrate-inducible *AYNI1* promoter, achieved a maximum catechol-1,2-dioxygenase activity of 280.6 U/L and 26.9 U/g of dry cell weight in yeast grown in minimal medium with nitrate as the nitrogen source and 1.5% glucose as the carbon source. In the same medium with glucose as the carbon source, catechol-1,2-dioxygenase activity was not detected for the control strain G1212/YIC102 with *ACDO1* expression under the regulation of its respective endogenous promoter. Gene expression analysis showed that *ACDO1* is induced by gallic acid and protocatechuic acid. In contrast to the wild-type strain, the *B. raffinosifermentans* strain with a deletion of the *ACDO1* gene was unable to grow on medium supplemented with gallic acid or protocatechuic acid as the sole carbon source. In summary, we propose that due to its substrate specificity, its thermal stability, and its ability to undergo long-term storage without significant loss of activity, *B. raffinosifermentans* catechol-1,2-dioxygenase (Acdo1p) is a promising enzyme candidate for industrial applications.

## Introduction

Petroleum products include far more than just fuels; they are important feedstocks for the chemical and pharmaceutical industry. To this day, there is a big competition between fuels and chemical feedstocks for a share of oil production. Many of these substances, like benzene, are considered harmful to the environment (Frumkin et al., 2009). Therefore, great emphasis is placed on research focusing on developing “green” synthetic approaches as alternatives to traditional chemical synthesis. In the spotlight are microbial chemical factories which convert biomass into desired feedstock product(s). However, because of its not well-defined composition, the conversion of biomass is still a challenge. Prior knowledge of the biochemical pathways of biomass deconstruction is just as important as the selection of the microorganisms involved and the definition of the culture conditions. *Escherichia coli* and *Saccharomyces* cerevisiae are among the best studied and most widely used microorganisms in the biotechnological industry. Multiple improved strains with modified metabolic pathways or newly introduced heterologous pathways are in commercial use. Nonetheless, the spectrum of compounds produced is still rather limited and the bioproduction of certain chemical compounds, like *cis,cis*-muconic acid, is still a challenge (Weber et al., 2012; Curran et al., 2013; Lin et al., 2014; Wang and Zheng, 2015; Brückner et al., 2018).

*Blastobotrys* strain LS3, which has been used in the past for a variety of characterizations and biotechnological experiments, was originally characterized by Gienow et al. (1990). Although this strain and its derivatives were long referred to as *Blastobotrys (Arxula) adeninivorans*, a recent study showed that LS3 could be reassigned to *Blastobotrys raffinosifermentans* (Thomas et al., 2019). This dimorphic, non-conventional yeast possesses many interesting features and metabolic pathways, including the degradation of *n*-butanol, purines, and tannins (Kunze et al., 2014; Malak et al., 2016; Thomas et al., 2019). Among the enzymes produced by *B. raffinosifermentans* LS3 is tannin acyl hydrolase 1 (Atan1p), catalyzing the first reaction step in tannin degradation. Expression of *ATAN1* is regulated by the carbon sources tannin or gallic acid (Böer et al., 2009a). The transformation of gallic acid and protocatechuic acid by the *B. raffinosifermentans* LS3 wild-type strain (Sietmann et al., 2010) was the first report of a non-oxidative decarboxylation of gallic acid by a eukaryotic microorganism. Cultivation experiments with protocatechuic and gallic acid suggested that the decarboxylation of protocatechuic and gallic acid may be catalyzed by the same enzyme (Sietmann et al., 2010). The authors also proposed a pathway for the transformation of hydroxylated benzoic acid derivatives by the LS3 strain, based on their observation that the metabolism of gallic acid leads to the production of pyrogallol and 2-hydroxymuconic acid, whereas the metabolism of protocatechuic acid produced catechol and *cis,cis*-muconic acid. The role of gallic acid decarboxylase (Agdc1p) in the tannic acid metabolic pathway was already investigated and described in Meier et al. (2017).

The goal of the present project was to investigate the role of catechol-1,2-dioxygenase in the tannin degradation pathway in the non-conventional yeast *B. raffinosifermentans*. Based on whole genome sequencing (Kunze et al., 2014), the annotated gene putatively encoding catechol-1,2-dioxygenase (ARAD1D18458g^1^) was selected and investigated. For this purpose, the amino acid sequence of the *ACDO1* gene product^2^ was compared with the amino acid sequences of catechol-1,2-dioxygenases known from other microbial sources. Based on their similarity, potentially conserved domains were identified and their presumed influence on activity of the active center as well as on enzyme stability was analyzed. The transformation/expression platform, Xplor^®^ 2 (Böer et al., 2009b), which allows rapid and simple genetic manipulations in *B. raffinosifermentans*, was used to construct recombinant strains overexpressing *ACDO1*. This recombinant enzyme was purified, its *in vitro* biochemical properties were characterized, and optimal working conditions established. In addition, a knock-out mutant lacking the *ACDO1* gene was created. Metabolite analysis of supernatants from the recombinant knock-out mutant as well as the control strain allowed the determination of the *in vivo* function and the enzymatic reactions carried out by this enzyme. Expression analyses of the *B. raffinosifermentans* wild-type strain LS3 shed light on the regulation of *ACDO1* as well as on the tannin degradation pathway. Finally, the microarray whole-genome gene expression dataset allowed the identification of transcripts and gene interactions involved in the catabolism of aromatic compounds. Based on these results, a map of the metabolic pathway for tannin degradation in *B. raffinosifermentans* described by Meier et al. (2017) was created. The findings reported in this paper are a contribution to the development of an innovative approach for the bioproduction of industrially relevant chemicals such as *cis,cis*-muconic acid, gallic acid, protocatechuic acid, catechol and pyrogallol by new microbial factories, as well as for the production of sought after enzymes.

## Materials and Methods

### Strains and Culture Conditions

*Escherichia coli* strains XL1 blue or DH5α were used as host strains for bacterial transformation and plasmid isolation (see Supplementary Table 1 for all strains and Supplementary Table 2 for all plasmids used in this study). All bacterial strains were cultured in Luria Bertani medium (LB – Sigma-Aldrich, St. Louis, MO, United States) supplemented with 50 mg/L ampicillin (AppliChem, Darmstadt, Germany) or 50 mg/L kanamycin (Carl Roth GmbH, Karlsruhe, Germany).

*Blastobotrys raffinosifermentans* LS3, wild-type strain (Kunze and Kunze, 1994), isolated from wood hydrolyzate in Siberia (Russia) and *B. raffinosifermentans* G1212 (*aleu2 ALEU2:atrp1*) (Steinborn et al., 2007) were used as experimental strains. The yeast strains were cultivated in shaking flasks at 30°C, 180 rpm either under non-selective conditions in complex medium (YEPD – yeast extract-peptone-dextrose) or in yeast minimal medium (YMM) supplemented with 43.5 mM NaNO_3_ as the nitrogen source and 20 g/L glucose as the carbon source (YMM-glucose-NaNO_3_) (Rösel and Kunze, 1995).

### Transformation and Isolation Procedures of Nucleic Acids

Transformation procedures of *E. coli* and *B. raffinosifermentans* were performed according to Böer et al. (2009a) and Bischoff et al. (2019). Plasmid DNA isolation and DNA restriction digests were carried out as described by Wartmann et al. (2002).

### Construction of *ACDO1* Expression Plasmids and Generation of Transgenic *Blastobotrys raffinosifermentans* Strains

For *ACDO1* overexpression, the *ACDO1* ORF with a 6His-tag encoding region at the 3′-end (*ACDO1-6H*) was amplified from genomic DNA of *B. raffinosifermentans* LS3 in a PCR reaction using primers that incorporated flanking *Eco*RI and *Not*I cleavage sites: primer ACDO1-1-6H – 5′- **GAA TTC** ATG GCC GGA TCA GGA CTT GAG GGC –3′ (primer nucleotide positions 1-24, *Eco*RI restriction site is in bold type and underlined); primer ACDO1-2-6H 5′-** GCG GCC GC**T TAG TGG TGG TGA TGA TGG TGG TCA AGG TCA GCG ATA GG –3′ (primer nucleotide positions 985–1005, 6His-tag encoding region underlined, *Not*I restriction site is in bold type and underlined). The resulting *Eco*RI-*Not*I flanked ACDO1-6H ORF was inserted into the plasmid pBS-AYNI1-PHO5-SS between the *B. raffinosifermentans* derived *AYNI1* promoter and the *S. cerevisiae PHO5* terminator (Böer et al., 2009c). The *AYNI1* promoter *– ACDO1-6H* gene *– PHO5* terminator flanked by *Spe*I *– Sac*II restriction sites expression modules were inserted into the plasmid Xplor2.2 to generate Xplor2.2-AYNI1-ACDO1-6H-PHO5 (Böer et al., 2009b). The resulting plasmids contained fragments of 25S rDNA, which are interrupted by the selection marker module (*ALEU2* promoter – *ATRP1m* gene – *ATRP1* terminator), the ACDO1 expression module and an *E. coli* resistance marker and replicator. To prepare the cassettes for yeast transformation, Xplor2.2-AYNI1-ACDO1-6H-PHO5 and the control plasmid Xplor2.2 lacking the ACDO1 expression module were digested with *Asc*I (YRC) or *Sbf*I (YIC) to remove the *E. coli* sequences including the resistance marker. The resulting restriction products YRC102-AYNI1-ACDO1-6H, YIC102-AYNI1-ACDO1-6H and YRC102 (control) were used to transform *B. raffinosifermentans* G1212.

Yeast transformants were selected by tryptophan auxotrophy in YMM-glucose-NaNO_3_. The cells were stabilized by passaging on selective (YMM-glucose-NaNO_3_) and non-selective (YEPD) agar medium, to attain a high level of protein production (Klabunde et al., 2003).

### Construction of a Δ*acdo1* Gene Disruption Mutant

To create disruption mutants, a gene disruption cassette containing a minimum of 500 bp upstream and downstream of the gene of interest as well as the ATRP1m selection marker module was constructed. All fragments were amplified by PCR using chromosomal DNA of *B. raffinosifermentans* LS3 as template and the ATRP1m selection marker module using plasmid pBS-ALEU2-TRP1m as the template (Steinborn et al., 2007). Primers used for fragment amplification were created with additional 15 bp overlaps (Supplementary Table 3). This strategy allowed ligation of fragments in one step by using an In-Fusion Cloning Kit (Takara Bio USA, San Jose, CA, United States). The resulting construct was amplified in *E. coli* DH5α. Finally, the complete construct covering an overlapping fragment upstream of the gene of interest, the *ATRP1m* selection marker module and an overlapping fragment downstream of the gene of interest was amplified using designed primers. The resulting product was amplified by PCR and used to transform *B. raffinosifermentans* G1212 (Böer et al., 2009a).

### Cell Growth Monitoring

#### OD_600nm_ Culture Measurement

For optical density measurement, a culture sample was diluted 1:10 or 1:100 to not exceed an OD_600nm_ value of 0.9, and 1 mL was pipetted in a 2 mL disposable plastic cuvette (BRAND GmbH, Wertheim, Germany), which was subsequently placed in a Jenway 7305 Spectrophotometer (Cole-Parmer, Stone, United Kingdom). Absorbance was measured at 600 nm. Experiments were performed in triplicate.

#### Determination of Dry Cell Weight

Two mL of yeast cultures were pipetted into pre-weighed 2 mL Eppendorf reaction tubes. Cells were pelleted by centrifugation at 25,000 *g* for 10 min at 4°C. Supernatants were discarded. Pellets were dried overnight in an oven at 80°C and the weight of the dried cell material was determined. Experiments were performed in triplicate.

### Determination of Catechol-1,2-Dioxygenase Activity

Various aromatic substrates were used to investigate the substrate specificity of catechol-1,2-dioxygenase. Prior to measurement, wavelength screening of reference compounds was performed to confirm the appropriate wavelengths from the literature. In addition, reaction products were scanned after each activity assay to exclude the formation of unexpected products. First, catechol-1,2-dioxygenase activity was assayed by following *cis,cis*-muconic acid formation from catechol or 2-hydroxymuconic acid formation from pyrogallol during the enzymatic reaction in 50 mM Tris-HCl buffer pH 7.5. Reactions were performed in 96-well UV-transparent microtiter plates (Greiner Bio-One GmbH, Frickenhausen, Germany) in a total volume of 200 μL. Experiments were done in triplicate. The reaction was started by adding 0.5 mM substrate solution (190 μL) to the enzyme (10 μL) and monitored 15 min at ∼20°C, 260 nm (ε = 16800 [1/M/cm]) and 296 nm (ε = 14500 [1/M/cm]) for *cis,cis*-muconic acid and 2-hydroxymuconic acid, respectively (Itoh, 1981). Blank values were established by using water instead of the enzyme. One unit (1 U) of enzyme activity was defined as the amount of enzyme required to oxidize 1 μmol catechol to *cis,cis*-muconic acid or 1 μmol pyrogallol to 2-hydroxymuconic acid per min at 20°C, pH 7.5.

To test the substrates hydroquinone, protocatechuic acid, gallic acid, 2,3-dihydroxybenzoic acid, and 2,5-dihydroxybenzoic acid and to assess potential 2,3-dioxygenase activity of the enzyme toward catechol, the assay conditions from the catechol assay were applied and product formation was tested at the wavelengths indicated in Section “Purification and characterization of Acdo1-6hp.”

Measurement of hydroxyquinol cleavage by catechol-1,2-dioxygenase was assayed by monitoring the reaction between hydroxyquinol and rhodanine which gives rise to a colored compound (modified method from Sharma et al., 2000). Absorbance was recorded against a water blank at 465 nm (ε = 42000 [1/M/cm]), and hydroxyquinol was used as the standard. One unit of catechol-1,2-dioxygenase activity was defined as the amount of enzyme required to release 1 mM 3-hydroxy-*cis,cis*-muconic acid per min at 20°C and pH 7.5. Activity levels were expressed as U/mg protein or as U/L. All measurements were done in triplicate and the *K*_M_ and V_max_ values for the substrates pyrogallol and catechol were determined from Lineweaver–Burk and Hanes–Woolf plots by linear regression using Microsoft Excel.

### Acdo1-6hp Molecular Mass Analysis

Acdo1-6hp was purified by column chromatography on a HisTrap FF (1 mL) column (Novagen, Madison, WI, United States) in 20 mM Tris, 0.5 mM NaCl (pH 7.9) and 5 mM imidazole as binding buffer and 20 mM Tris, 0.5 mM NaCl (pH 7.9) and 1 M imidazole as elution buffer. Finally, the purified protein was desalted using PD10 columns (GE Healthcare Europe GmbH, Freiburg, Germany).

The indicative molecular mass determination of native Acdo1-6hp was done by gel filtration using Superdex™ 200 columns (GE Healthcare, Amersham, United Kingdom). The flow rate was 1 mL/min and fractions of about 1 mL were collected for 182 min [buffer: 50 mM Tris (pH 8) and 0.15 M NaCl]. A calibration curve was constructed using blue dextran, ferritin, catalase, bovine albumin, RNAse A and vitamin B12 as standards.

### Protein Analysis

Sodium dodecyl sulfate-polyacrylamide gel electrophoresis (SDS-PAGE) and Western blot analyses were performed as described by Kunze et al. (1998). The antibodies used for Western blot analysis were anti-His-tag specific primary antibody produced in rabbit (Sigma-Aldrich, St. Louis, MO, United States) and secondary antibody anti-mouse IgG alkaline phosphatase conjugate produced in goat (Sigma-Aldrich, St. Louis, MO, United States). The staining procedure was done by membrane incubation with nitro-blue tetrazolium chloride/5-bromo-4-chloro-3-indolyl phosphate (NBT/BCIP) substrate (Roche Diagnostics, Rotkreuz, Switzerland).

The dye-binding method of Bradford was used for protein quantification (Bio-Rad, Hercules, CA, United States) using bovine serum albumin as the standard (Bradford, 1976).

### Nested Quantitative Real-Time PCR

For analysis of temporal gene expression patterns, a nested qRT-PCR assay was applied (Worch and Lemke, 2017). The first PCR was performed with cDNA-template (10 cycles) using gene-specific primers and RTA-1 primer (Supplementary Table 4). The PCR product was diluted 1:500 and amplified in the presence of SYBR^®^ Green fluorescent dye (Power SYBR Green PCR Master Mix, Applied Biosystems, Foster City, CA, United States) using the ABI 7900HT Fast Sequence Detection System (Applied Biosystems) with gene-specific primers and RTA primer. *TFCI* and *ALG9* were used as reference (housekeeping) genes (Rösel and Kunze, 1995; Teste et al., 2009). Calculations were done using the ΔΔct-method (Livak and Schmittgen, 2001).

### Microarray Design and Hybridization for Gene Expression Analysis

Gene expression profiling was performed using a microarray produced by Agilent Technologies in 8 × 60 k format. The microarray was based on 6,025 annotated chromosomal sequences and 36 putative mitochondrial gene oligos and was designed using Agilent Technologies eArray software^3^ (design number 035454). Depending on sequence length of genes, up to ten 60-mers per gene were created, resulting in a total of 56,312 *B. raffinosifermentans* specific oligos. *B. raffinosifermentans* LS3 was cultivated overnight in YMM-glucose-NaNO_3_. Cells were pelleted by centrifugation for 5 min at 3,220 × *g*, 20°C and shifted to YMM-NaNO_3_ containing a mixture of 0.5% protocatechuic acid plus 1% glucose or 1% glucose only. After 15 min, 30 min, 2 h and 5 h of cultivation at 30°C and 180 rpm, cells were harvested, and total RNA was isolated using the RNeasy Mini Kit (Qiagen GmbH, Hilden, Germany). Samples were labeled and the microarray hybridized according to the Agilent Technologies “One-Color Microarray-Based Gene Expression Analysis (v6.5)” instructions. R package limma was used for microarray data analysis (Smyth, 2005). Background of raw expression data was corrected by “normexp” and normalized between arrays using “quantile.” Differentially expressed genes were detected by fitting a linear model to log2-transformed data by an empirical Bayes method (Smyth, 2005). The Bonferroni method was used to correct for multiple testing (Bonferroni, 1936).

### Metabolite Analysis by Gas Chromatography – Mass Spectrometry

#### Sample Collection, Extraction and Derivatization Procedure

Quantitative measurements of hydroxylated aromatic acids, as well as various phenol derivatives, were performed by Gas Chromatography – Mass Spectrometry (GC-MS) (Clarus SQ 8 GC Mass Spectrometer). Prior to this, samples were extracted and derivatized. For this purpose, 2 mL yeast culture was centrifuged for 10 min, 16,000 *g*, 4°C. Supernatant was collected in fresh Eppendorf reaction tubes and pellet was discarded. Five hundred μL supernatant was adjusted to pH 2 with HCl and compounds were extracted with 2 × 300 μL and 1 × 400 μL MTBS (Sigma-Aldrich, St. Louis, MO, United States). The upper extract layer was collected in fresh reaction tubes. Extracts were lyophilized in a Freeze Dryer, Alpha 1-4 LSC plus (Christ) and resuspended in 400 μL pyridine. After adding 100 μL BSTFA (Sigma-Aldrich, St. Louis, MO, United States) samples were incubated overnight at 60°C.

#### Gas Chromatography – Mass Spectrometry Measurement

A 1 μL aliquot of sample was injected into an Elite-5MS column, Length 30 m, I.D 0.25 mm, film thickness 0.25 μm (PerkinElmer LAS, Rodgau, Germany). The injection was performed with a split flow rate of 10 mL/min. Temperature was kept at 60°C for 1 min then ramped up to 230°C at 15°C/min and held at 230°C for 11.33 min. Peak areas were calculated by TurboMass 6.1 using data from external quantification standards assayed four times. Experiments were performed in triplicate.

## Results

### Identification of a *Blastobotrys raffinosifermentans ACDO1* Gene Encoding for Catechol-1,2-Dioxygenase (Acdo1p)

A putative catechol-1,2-dioxygenase gene (ARAD1D18458g) was annotated in the genome of *B. raffinosifermentans* LS3. The gene, Arxula catechol-1,2-dioxygenase 1 (*ACDO1*), is localized on chromosome *Arad1D*, position 1512067–1513071. The 1005 bp open reading frame contains no introns and encodes a protein with 335 amino acids. The predicted subunit molecular mass of Acdo1p is 37.76 kDa. Protein analysis according to the SignalP program (version 4.1^4^) predicts the absence of a secretion signal sequence.

**FIGURE 1:**
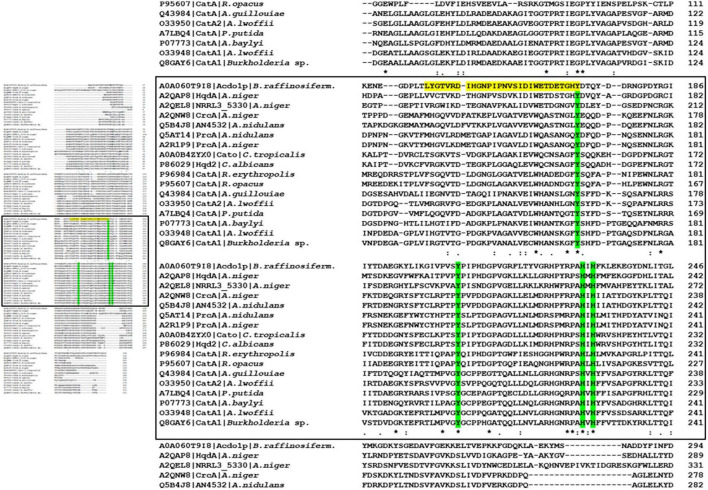
Sequence alignment of bacterial and fungal intradiol ring cleavage dioxygenases. **(Left panel)** Overview of the alignment. Complete data are available in Supplementary Figure 10. **(Left panel)** Magnification of the regions with predicted catalytic domain and metal binding sites. The Acdo1p amino acid sequence was aligned to bacterial and fungal intradiol ring cleavage dioxygenases of *A. niger* (UniProt accession numbers A2QAP8, A2QEL8, A2QNW8, A2R1P9), *A. nidulans* (Q5B4J8, Q5AT14), *C. tropicalis* (A0A0B4ZYX0), *C. albicans* (P86029), *A. baylyi* (P07773), *A. lwoffii* (O33948), *A. lwoffii* (O33950), *A. guillouiae* (Q43984), *R. erythropolis* (P96984), *R. opacus* (P95607), *Burkholderia* sp. (Q8GAY6), and *P. putida* (A7LBQ4) from the UniProt database. Alignment was done using ClustalOmega software (Sievers et al., 2011, http://www.ebi.ac.uk/Tools/msa/clustalo/). Residues marked with an asterisk (*) are identical in all sequences in the alignment. A colon (:) indicates conserved substitutions and a dot (.) indicates semi-conserved substitutions. The predicted catalytic domain is highlighted in yellow and metal binding sites are in green.

**FIGURE 2:**
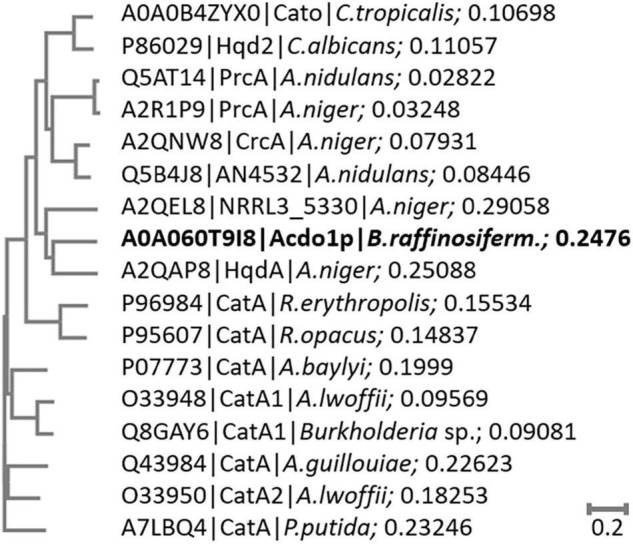
Phylogenetic tree of bacterial and fungal intradiol ring cleavage dioxygenases. The phylogenetic tree was constructed by the neighbor joining method of Acdo1p to selected bacterial and fungal dioxygenase amino acid sequences without distance corrections. The length of the branches is indicative of the evolutionary distance between the sequences. The distance values show the number of substitutions (amino acid residues) as a proportion of the length of the alignment (excluding gaps). Larger numbers represent a larger amount of genetic change.

Sequence alignment of Acdo1p with known intradiol ring cleavage dioxygenases revealed multiple conserved domains that are fundamental for enzyme catalytic activity and structure, including the highly conserved metal-binding sites Tyr171, Tyr205, His229, His231 (Figure 1). These conserved residues in Acdo1p, however, do not specifically prove that Acdo1p is a catechol-1,2-dioxygenase, due to the fact that they are found in other dioxygenases, too (e.g., in the intradiol ring-cleavage dioxygenase PrcA from *Aspergillus nidulans*). The Acdo1p sequence was therefore compared with other known catechol-1,2-dioxygenases. An alignment of Acdo1p with the catechol-1,2-dioxygenase Hqd2 (P86029) from *Candida albicans* resulted in 30.2% identity with 102 identical positions and 113 similar positions. HqdA (A2QAP8) from *A. niger* is even more similar: 49.4% identity with 165 identical positions and 95 similar positions, suggesting that Acdo1p is indeed a catechol-1,2-dioxygenase. Additionally, in order to address evolutionary distances and the amount of genetic change, a phylogenetic tree between Acdo1p and various bacterial and fungal intradiol ring cleavage dioxygenases was made using the neighbor joining method (Figure 2). Crystallographic studies have shown that catechol-1,2-dioxygenases are homodimers which incorporate a non-heme ferric iron atom (Fe^3+^) as cofactor. A catecholate substrate binds to the Fe atom by means of the deprotonated hydroxyl groups. The iron abstracts an electron to produce a substrate radical. This then allows the reaction with dioxygen and a subsequent intradiol cleavage to occur.

### Generation of Recombinant *Blastobotrys raffinosifermentans* Strains

*Blastobotrys raffinosifermentans* G1212 [Δ*trp1*] was used to construct an Acdo1-6hp producing yeast strain. A 6His-tag encoding sequence was fused to the 3′-end of the ORF of the *ACDO1* gene under control of the nitrate inducible *AYNI1* promoter (*B. raffinosifermentans* nitrate reductase promoter). Cassettes with the *ACDO1* expression module (YRC102-AYNI1-ACDO1-6H, YIC102-AYNI1-ACDO1-6H) (Supplementary Figure 1) and controls (YRC102, YIC102) were prepared as described in Materials and Methods Section “Construction of *ACDO1* Expression Plasmids and Generation of Transgenic *B. raffinosifermentans* Strains.” After genome integration, selected clones (YICs and YRCs) were passaged to establish high plasmid stability. After cultivation in YMM-glucose-NaNO_3_ at 30°C for 48 h, cells were harvested and screened for catechol-1,2-dioxygenase activity. This activity was present in all stabilized *B. raffinosifermentans* G1212/YRC102-AYNI1-ACDO1-6H and G1212/YIC102-AYNI1-ACDO1-6H strains.

**FIGURE 3:**
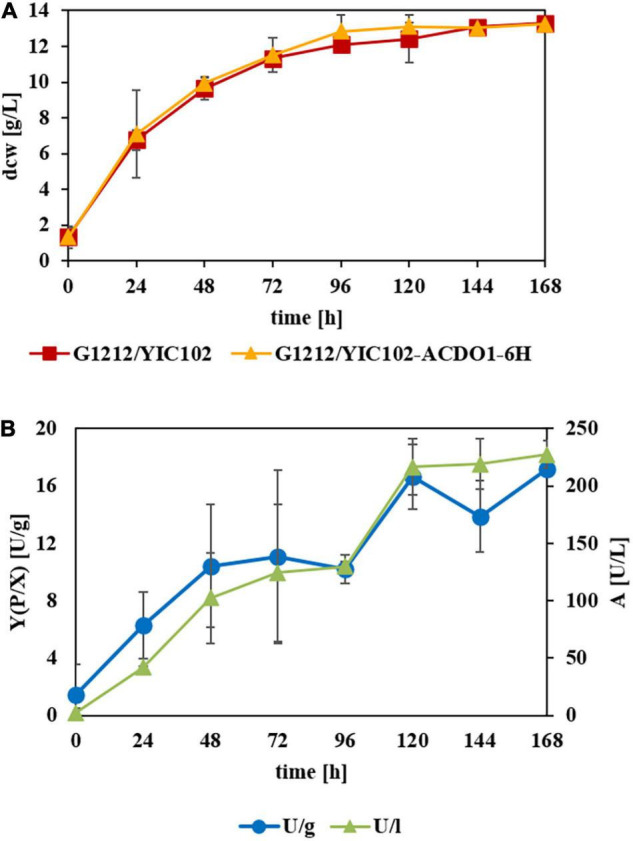
Time-course analysis of catechol-1,2-dioxygenase activity in *B. raffinosifermentans* control strain and strain with overexpression of ACDO1-6H. The cells were grown on YMM-glucose-NaNO_3_ in shaking flasks at 30°C for 168 h. **(A)** Measurement of cell growth. **(B)** Intracellular Acdo1-6hp activity (triangles) and calculated yield [Y(P/X)] (Y – yield coefficient, P – activity in U, X – biomass in g) (circles) for G1212/YIC102-ACDO1-6H.

Since the best transgenic strains belonged to the genome-integrated YICs, the *B. raffinosifermentans* strains G1212/YIC102 and G1212/YIC102-AYNI1-ACDO1-6H were then cultivated in YMM-glucose-NaNO_3_ at 30°C and 180 rpm for 168 h, in order to follow a time course of enzymatic activity. Dry cell weight (dcw), catechol-1,2-dioxygenase activity with catechol as the substrate and yield [Y(P/X)] (Y – yield coefficient, P – activity in U, X – biomass in g) were determined once every 24 h. Catechol-1,2-dioxygenase activity was detectable in the disrupted cells of the selected transgenic strain (*B. raffinosifermentans* G1212/YIC102-AYNI1-ACDO1-6H) but not in the control strain *B. raffinosifermentans* G1212/YIC102 when glucose served as the sole carbon source. Both yeast strains reached a maximum dcw after 72 h of cultivation and then biomass remained constant until the end of the experiment. The transgenic strain *B. raffinosifermentans* G1212/YIC102-AYNI1-ACDO1-6H achieved its maximum enzyme activity of ≈ 220 U/L and ≈ 17 U/g dcw after 120 h (Figure 3). The wild-type strain G1212/YIC102 did not exert Acdo1 activity (data not shown).

To analyze the function of *ACDO1*, the gene was deleted by homologous recombination using *B. raffinosifermentans* G1212 [Δ*trp1*] as a host strain. To avoid affecting any possible nearby genes, the deletion mutant cassette was constructed using 381 bp of the upstream homology fragment + 142 bp of the 5′ gene coding sequence and 158 bp of the downstream homology fragment + 361 bp of the 3′ gene coding sequence (Supplementary Figure 2). After the selection marker indicated integration of the cassette, the correct integration locus was verified by PCR using primers (listed in Supplementary Table 3) amplifying part of the integrated marker and an adjacent area of genomic DNA (Supplementary Figure 3). The strain with a correctly integrated deletion cassette was labeled G1235 [Δ*trp1*].

### Purification and Characterization of Acdo1-6hp

*Blastobotrys raffinosifermentans* G1212/YIC102-AYNI1-ACDO1-6H with a 6His-tag-encoding sequence at the 3′-end of the *ACDO1* ORF was selected for synthesis of recombinant Acdo1-6hp. Purification was done by Immobilized Metal Affinity Chromatography (IMAC) using an ÄKTA Pure System on HisTrap HP His-tag protein purification columns (Cytiva Europe GmbH, Freiburg, Germany). Acdo1-6h was eluted at approximately 860 mM imidazole. Acdo1-6hp has a predicted molecular mass of 37.76 kDa. The denatured protein in both crude extract and the purified protein fraction was visible at a position of 40 kDa on SDS-PAGE and Western blot (Supplementary Figure 4A).

Catechol-1,2-dioxygenase total activity was 0.92 ± 0.02 U in crude extract and 0.85 ± 0.02 U in the purified fraction, with a specific activity of 0.82 ± 0.02 U/mg for the crude extract and 5.39 ± 0.13 U/mg for the purified fraction. Purification yield was approximately 93% (Table 1).

**TABLE 1:** Summary of Acdo1-6hp purification.

Step	Protein [mg]	A_*total*_ [U]	A_*specific*_ [U/mg]	Yield [%]	Purification fold
Crude extract	1.13 ± 0.01	0.92 ± 0.2	0.82 ± 0.02	100	1
Purified protein	0.16 ± 0.01	0.85 ± 0.02	5.39 ± 0.13	92.62	6.61

*The protein was purified from 200 mL culture incubated in shaking flasks for 48 h at 30°C and 180 rpm. Values are shown as mean ± one standard deviation.*

The molecular mass of Acdo1-6hp was estimated by gel filtration on a Superdex™ 200 column and compared to a standard curve using molecules with known molecular masses (Blue Dextran – 20,000,000 Da, Ferritin – 450,000 Da, Catalase – 240,000 Da, BSA – 67,000 Da, RNAse A – 13,700 Da and Vitamin B12 – 1355 Da). Accordingly, the native enzyme had a calculated mass of 37.8 kDa. Since the predicted molecular mass of the 6His-tagged Acdo1-6hp is 79.7 kDa, this indicates that Acdo1-6hp is a dimeric protein.

The optimal pH conditions for Acdo1-6hp activity were established *in vitro* using 0.5 mM catechol as the substrate and the following buffers: 50 mM sodium citrate (pH 2.5 – 6.5), 50 mM sodium acetate (pH 3.5– 6), 50 mM sodium phosphate (pH 5.5 – 8) and 50 mM TRIS-HCl (pH 7 – 9). The highest relative enzyme activity of 80% and more was observed in the pH range of 7.0 – 8.0 with an absolute peak (100%) at pH 7.5 (Supplementary Figure 4B).

The effect of temperature on the activity of catechol-1,2-dioxygenase was measured by incubating the reaction mixture at different temperatures under optimal pH conditions. Relative enzyme activity in excess of 80% was observed at temperatures between 21 and 39°C (Supplementary Figure 4B). Acdo1-6hp was most active at 25°C. Acdo1-6hp is stable between 4 and 30°C, shown by the fact that enzyme activity after 23 h of incubation at these temperatures remained at above 85% of the initial value (Supplementary Figure 5). Increasing the temperature to 40°C resulted in a decrease of enzyme activity to 85% after 7 h and complete loss of activity after 23 h. At 50°C, enzyme activity fell to 60% within 1 h and was completely abolished after 7 h of incubation. In a crude extract kept at 4°C, Acdo1-6hp enzyme activity rapidly declined with time, showing 50 and 60% loss after 24 and 48 h of storage, respectively. After 55 days of storage, no enzyme activity was detected in the crude extract. In contrast to the crude extract, enzyme activity in the purified fraction remained far more stable, still exhibiting 99% of initial activity after 55 days at 4°C. Freezing samples in liquid nitrogen resulted in complete loss of Acdo1-6hp activity in both the crude extract and purified fraction.

To identify possible inhibitors and/or cofactors of Acdo1-6hp, the enzyme was incubated in reaction mixtures containing a variety of supplements at 1 mM final concentration. Strong enzyme inhibition (relative activity < 30%) was observed in the presence of Ni^2+^, Al^3+^, Zn^2+^, PEG4000, PEG6000, PEG8000, or EDTA. In contrast, addition of Mg^2+^, Ca^2+^, and Fe^3+^ to the reaction mixture had no significant influence on catechol-1,2-dioxygenase activity (relative activity > 80%). During the experiments it was observed that addition of cOmplete™ EDTA-free Protease Inhibitor Cocktail (Roche, Penzberg, Germany) to the lysis buffer during the purification procedure caused an almost complete inhibition of Acdo1-6hp activity (Table 2).

**TABLE 2:** Effect of metal salts and additives on Acdo1-6hp activity.

1 [mM]	Relative activity [%]	1 [mM]	Relative activity [%]	1 [mM]	Relative activity [%]
control (H_2_O)	100 ± 4	ZnSO_4_	5 ± 3	PEG4000	12 ± 2
NiSO_4_	19 ± 2	ZnCl_2_	14 ± 2	PEG6000	11 ± 2
NiCl_2_	25 ± 1	AlCl_3_	0 ± 48	PEG8000	13 ± 2
FeSO_4_	46 ± 3	MgSO_4_	84 ± 12	EDTA	12 ± 4
FeCl_3_	98 ± 7	MgCl_2_	108 ± 13	cOmplete™, EDTA-free Protease Inhibitor Cocktail	6 ± 3
CoSO_4_	48 ± 3	MnSO_4_	80 ± 5		
CoCl_2_	63 ± 6	MnCl_2_	37 ± 9		
CuSO_4_	38 ± 8	CaSO_4_	105 ± 5		
CuCl_2_	55 ± 7	CaCl_2_	103 ± 1		

*Relative Acdo1-6hp activity [%] was analyzed in the presence of 0.5 mM catechol as the substrate. Values are shown as mean ± one standard deviation.*

The kinetic parameters of purified Acdo1-6hp were determined photometrically using pyrogallol, catechol and hydroxyquinol as substrates. The *K*_M_ of Acdo1-6hp for pyrogallol (0.1 ± 0.02 mM) was found to be 25 times higher than for catechol (0.004 ± 0.001 mM) and 12.5 times higher than for hydroxyquinol (0.008 ± 0.002 mM). Turnover (*k*_cat_) was 10.6 ± 0.4 s^–1^ for pyrogallol, 15.6 ± 0.4 s^–1^ for catechol, and 17.0 ± 3.5 s^–1^ for hydroxyquinol. With a value of 119 ± 18 [mM^–1^ s^–1^] the catalytic efficiency for pyrogallol was found to be much lower than for catechol (3950 ± 930 [mM^–1^ s^–1^]) and for hydroxyquinol (2130 ± 971 [mM^–1^ s^–1^]) (Table 3 and Supplementary Figure 6).

**TABLE 3:** Kinetic constants of purified Acdo1-6hp using pyrogallol, catechol or hydroxyquinol as substrates.

Substrate	*K*_*M*_ [mM]	*k*_*cat*_ [s^–1^]	*k*_*cat*_/*K*_*M*_ [mM^–1^s^–1^]
Pyrogallol	0.1 ± 0.02	10.6 ± 0.4	119 ± 18
Catechol	0.004 ± 0.001	15.6 ± 0.4	3950 ± 930
Hydroxyquinol	0.008 ± 0.002	17.0 ± 3.5	2130 ± 971

*Values are shown as mean ± one standard deviation.*

Several substrates differing in the number and positions of hydroxyl groups were tested to determine the *in vitro* substrate specificity of Acdo1-6h. The enzymatic reactions were carried out under optimal conditions for catechol-1,2-dioxygenase. Reactions were monitored spectrophotometrically under different wavelengths depending on the tested substrates (Table 4). The results not only showed the specificity of Acdo1-6hp for pyrogallol, catechol and hydroxyquinol but also confirmed that Acdo1-6hp is a catechol-1,2-dioxygenase but not a catechol-2,3-dioxygenase. The reaction carried out for the catechol-2,3-dioxygenase activity (monitored at a wavelength of 375 nm) failed to show formation of 2-hydroxymuconic semialdehyde as the expected product. Evidently, the positions of hydroxyl groups on the aromatic ring are crucial for the enzymatic reaction by Acdo1-6hp. Substrates with hydroxyl groups at position 1 and 2 are accepted by the enzyme, regardless of whether there is no additional hydroxyl group (catechol), an additional one at the meta position (pyrogallol) or one at the para position (hydroxyquinol). However, the presence of a carboxyl group appears to have a strong effect on substrate binding, since protocatechuic acid, 2,3-dihydroxybenzoic acid, and gallic acid, despite the same location of the hydroxyl groups, fail to be converted by Acdo1p-6hp.

**TABLE 4:** Spectrophotometric substrate analysis of Acdo1-6hp.

Substrate	Product	λ	U_*sp*_ [U g^–1^]
Catechol	*Cis,cis*-muconic acid	260 nm	0.57 ± 0.03
Catechol	2-Hydroxymuconic semialdehyde	375 nm	0.00 ± 0.00
Hydroquinone	4-Hydroxymuconic acid semialdehyde	320 nm	0.00 ± 0.00
Hydroxyquinol	3-Hydroxy-*cis,cis*-muconic acid	465 nm	2.78 ± 0.49
Pyrogallol	2-Hydroxymuconic acid	296 nm	0.59 ± 0.01
Protocatechuic acid	3-Carboxy-*cis,cis*-muconate	290 nm	0.02 ± 0.00
Gallic acid	4-Carboxy-2-hydroxymuconic semialdehyde	259 nm	0.02 ± 0.05
2,3-Dihydroxybenzoic acid	3-Carboxy-2-hydroxymuconate semialdehyde	268 nm	0.00 ± 0.00
2,5-Dihydroxybenzoic acid	Maleyl pyruvate	334 nm	0.00 ± 0.03

*Values are shown as mean ± one standard deviation.*

### Expression Analysis of *ACDO1* on Various Carbon Sources

**FIGURE 4:**
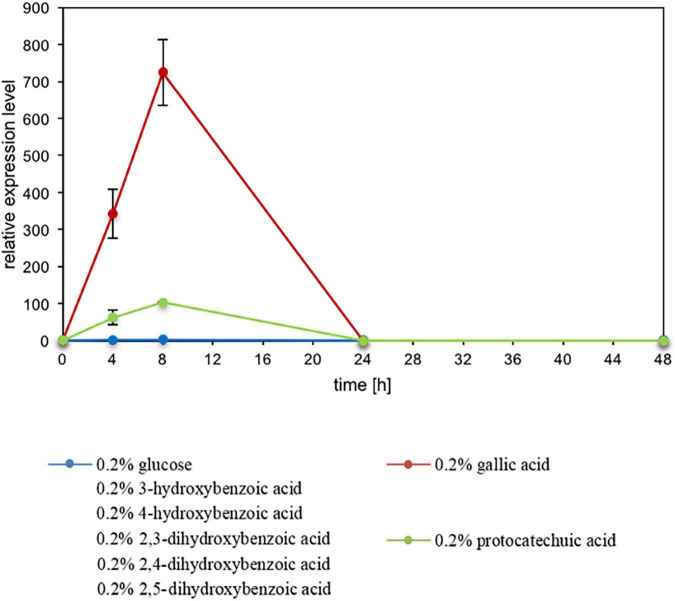
Influence of carbon sources on *ACDO1* expression. Gene expression of *ACDO1* was analyzed in *B. raffinosifermentans* G1212/YIC102 cultivated on YMM-NaNO_3_ supplemented with 0.2% of either glucose, gallic acid, protocatechuic acid, 3-hydroxybenzoic acid, 4-hydroxybenzoic acid, 2,3-dihydroxybenzoic acid, 2,4-dihydroxybenzoic acid or 2,5-dihydroxybenzoic acid as the sole source of carbon. Quantitative real time PCR analysis revealed that only gallic acid and protocatechuic acid supply leads to increased expression of the *ACDO1* gene. Error bars represent one standard deviation.

The expression level of *ACDO1* relative to the housekeeping gene *TFCI* was analyzed by nested quantitative RT-PCR. Primers used for the expression analysis of *ACDO1* are shown in Supplementary Table 4. *B. raffinosifermentans* G1212/YIC102 was cultivated on YMM-NaNO_3_ supplemented with 0.2% (w/v) of either glucose or different hydroxylated aromatic acids as the sole source of carbon. Samples were collected at different times and total RNA was isolated. An increase of gene transcription of *ACDO1* was observed when cells were cultivated on gallic acid and protocatechuic acid. The highest expression level was detected after 8 h of cultivation (Figure 4). In contrast, none of the other carbon sources (hydroxyquinol, glucose, 3-hydroxybenzoic acid, 4-hydroxybenzoic acid, 2,4-dihydroxybenzoic acid, 2,3-dihydroxybenzoic acid, 2,5-dihydroxybenzoic acid) altered the expression of *ACDO1*. In order to search for identities between the *ACDO1* promoter and that of the gallic acid decarboxylase 1 gene (*AGDC1*) from *B. raffinosifermentans* (Meier et al., 2017), the genomic sequences of ARAD1D18458g and ARAD1C45716g were retrieved from the GRYC database^5^ and extended to 600 bp upstream of the genes. Sequence alignment using the Blastn suite (Altschul et al., 1997) revealed no significant similarity (data not shown).

To evaluate whole transcriptome variations upon incubation of *B. raffinosifermentans* with protocatechuic acid, microarray expression analysis was performed. Changes in gene expression of the wild-type strain, *B. raffinosifermentans* LS3, cultivated in YMM-NaNO_3_ supplemented with 1% glucose or YMM-NaNO_3_ supplemented with 1% glucose plus 0.5% protocatechuic acid were analyzed over time. For this, cells were harvested after 15, 30 min, 2 and 5 h of incubation in shaking flasks at 30°C and 180 rpm. After RNA isolation, microarray data were used to identify main candidate genes, that may be involved in the degradation of protocatechuic acid. In order to learn which genes could potentially be responsible for protocatechuic acid degradation, the microarray data were searched in particular for those genes whose expression was strongly increased by protocatechuic acid supply and whose annotations or gene descriptions indicated hydroxylase activity. In this regard, we identified ARAD1C03498g, ARAD1D18392g, ARAD1D27984g, ARAD1C08580g, ARAD1C00330g, and ARAD1C08558g as the most promising candidates, with ARAD1C00330g showing the highest expression change. Significant upregulation was also observed for genes encoding gallic acid decarboxylase 1 (*AGDC1*), catechol-1,2-dioxygenase (*ACDO1*) and tannase 1 (*ATAN1*) as well as for genes annotated as putative tannase 2 (*ATAN2*), oxalocrotonate decarboxylase, 2-oxopent-4-enoate hydratase and 4-hydroxy-2-oxovalerate aldolase (Supplementary Figure 7). These data suggest that additional enzymes are involved in tannic acid degradation. In Supplementary Figure 8, an alternative protocatechuic acid degradation pathway is presented based on data from these microarray experiments and gene annotations available in databases. Supplementary Table 5 provides an overview of all open reading frames identified in this study and compares them to known genes with respect to sequence identities.

### Role of Acdo1p in *Blastobotrys raffinosifermentans* in Metabolizing Protocatechuic Acid

**FIGURE 5:**
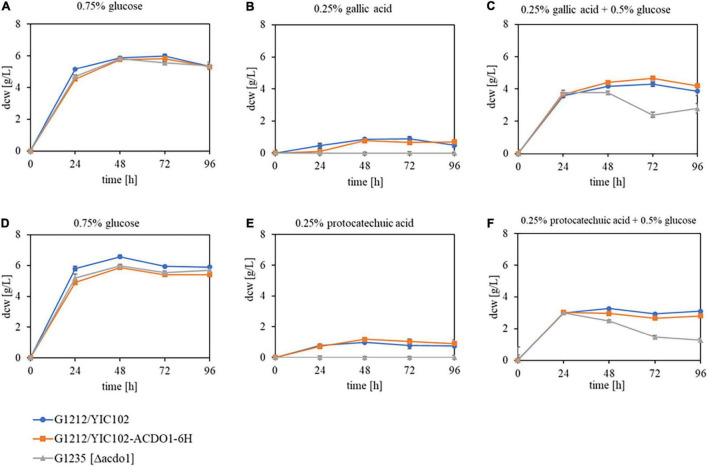
Growth behavior of *B. raffinosifermentans* strains G1212/YIC102 (control), G1212/YIC102-AYNI1-ACDO1-6H (ACDO1-6H overexpression) and G1235 [Δ*acdo1*] (*ACDO1* deletion). Strains were cultivated on HMM-NO_3_ supplemented with **(A,D)** 0.75% glucose, **(B)** 0.25% gallic acid, **(C)** 0.25% gallic acid + 0.5% glucose, **(E)** 0.25% protocatechuic acid, and **(F)** 0.25% protocatechuic acid + 0.5% glucose as the sole source of carbon. Error bars represent one standard deviation.

The enzyme Acdo1p is, similar to *B. raffinosifermentans* gallic acid decarboxylase 1, encoded by an inducible gene. The induction occurs in the presence of gallic acid as well as protocatechuic acid. The analysis of its *in vivo* role was enabled by the generation of the *B. raffinosifermentans* strains G1212/YIC102, G1212/YIC102-AYNI1-ACDO1-6H and G1235 [Δ*acdo1*]. The strains were cultivated at 30°C and 180 rpm on YMM-NaNO_3_ supplemented with 0.75% glucose, 0.25% gallic acid, 0.25% gallic acid + 0.5% glucose, 0.25% protocatechuic acid, and 0.25% protocatechuic acid + 0.5% glucose (all values in w/v) as the sole sources of carbon and energy. Growth behavior was monitored by dcw measurement (Figure 5). All strains performed best in medium supplemented with glucose as the sole source of carbon. Growth on glucose was not affected by the deletion of *ACDO1* (Figures 5A,D). Partial substitution of glucose with gallic acid (Figure 5C) led to a slight reduction of cell growth in all strains, e.g., at time-point 48 h, a dcw reduction of 24% was observed in the *ACDO1* overexpressing strain compared to growth on glucose as sole substrate (Figure 5A). Even more so when glucose was partially substituted with protocatechuic acid (Figure 5F), growth reduction of up to 50% was seen compared to growth on glucose alone (Figure 5D, *ACDO1* strain at 48 h, 5.87 g/L vs. 2.96 g/L). Notably, growth of the Δ*acdo1* deletion strain G1235 started to drop after 24 h (in 0.25% gallic acid and 0.5% glucose, Figure 5C) or 48 h, respectively (in 0.25% protocatechuic acid and 0.5% glucose, Figure 5F), whereas growth of control strain and overexpression strain remained rather constant. Weakest growth was observed in medium supplemented with 0.25% gallic acid or 0.25% protocatechuic acid as the sole source of carbon (Figures 5B,E). Under these conditions, growth of the *ACDO1* deletion strain G1235 [Δ*acdo1*] was completely inhibited.

**FIGURE 6:**
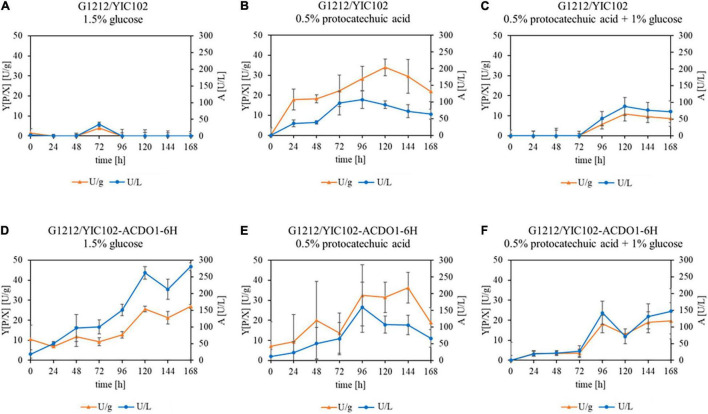
Acdo1-6hp activity in *B. raffinosifermentans* strains G1212/YIC102 (control) and G1212/YIC102-ACDO1-6H (ACDO1-6H overexpression). The diagrams show the results of in vitro activity assays with crude extract of **(A–C)** control and **(D–F)** ACDO1-6H overexpressing strains. Cells were harvested every 24 h after cultivation on YMM-NaNO_3_ with 1.5% glucose, 0.5% protocatechuic acid, or 1% glucose plus 0.5% protocatechuic acid as the carbon source, were disrupted using a bead mill, and crude extract was used under the assay conditions described in Section “Determination of Catechol-1,2-Dioxygenase Activity” with catechol as the substrate. Error bars represent one standard deviation.

In a time course experiment, variations in catechol-1,2-dioxygenase activity and yield were measured for the control strain G1212/YIC102 and the ACDO1-6H overexpressing strain G1212/YIC102-ACDO1-6H after cultivation on various carbon sources. Both strains were cultivated at 30°C in shaking flasks on YMM-NaNO_3_ supplemented with 1.5% glucose, 0.5% protocatechuic acid or 0.5% protocatechuic acid + 1% glucose for up to 168 h (Figure 6). Enzyme activity was measured every 24 h *in vitro* using catechol as the substrate under the assay conditions described in Section “Determination of Catechol-1,2-Dioxygenase Activity,” with the modification that instead of pure enzyme, crude extract of the cells was used, obtained by disrupting the cells using a bead mill. In the presence of glucose as the sole carbon source, catechol-1,2-dioxygenase activity was virtually absent from crude extract of *B. raffinosifermentans* G1212/YIC102 (Figure 6A) but was prominently present and showing a strong increase in time in strain G1212/YIC102- ACDO1-6H ultimately reaching 280.6 U/L and 26.9 U/g of dry cell weight (Figure 6D). When protocatechuic acid was the sole carbon source, both control strain G1212/YIC102 and overexpressing strain G1212/YIC102-ACDO1-6H showed a similar strong increase of catechol-1,2-dioxygenase activity with time (Figures 6B,E). In medium containing 0.5% protocatechuic acid and 1% glucose, enzyme activity initially remained low before a distinct increase after 72 h of incubation (Figures 6C,F). Whereas in the control strain enzyme activity reached 76 U/L, in the overexpressing strain it reached 150 U/L.

### Metabolite Analysis During Degradation of Gallic Acid and Protocatechuic Acid by *Blastobotrys raffinosifermentans*

**FIGURE 7:**
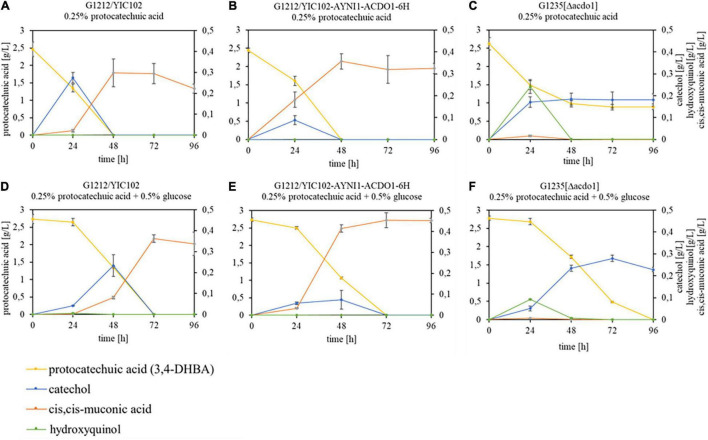
GC-MS analysis of substrate – product conversion during cultivation of *B. raffinosifermentans* on protocatechuic acid. **(A,D)** *B. raffinosifermentans* G1212/YIC102; **(B,E)** *B. raffinosifermentans* G1212/YIC102-AYNI1-ACDO1-6H; **(C,F)** *B. raffinosifermentans* G1235 [Δ*acdo1*]. Strains were cultivated on HMM-NaNO_3_ supplemented with 0.25% protocatechuic acid **(A–C)** or with 0.25% protocatechuic acid plus 0.5% glucose **(D–F)**. Protocatechuic acid concentration is displayed on the left *y*-axis; catechol, *cis,cis*-muconic acid and hydroxyquinol concentrations are displayed on the right *y*-axis. Error bars represent one standard deviation.

To investigate the *in vivo* transformation of protocatechuic acid, *B. raffinosifermentans* strains G1212/YIC102 (control), G1212/YIC102-AYNI1-ACDO1-6H (overexpression of *ACDO1- 6H*) and G1235 [Δ*acdo1*] were cultivated on HMM-NaNO_3_ supplemented with 0.25% protocatechuic acid or with 0.25% protocatechuic acid + 0.5% glucose (Figure 7). In the presence of protocatechuic acid as the sole source of carbon, control and G1212/YIC102-AYNI1-ACDO1-6H strain achieved complete consumption of protocatechuic acid within 48 h of cultivation while consumption in the deletion strain G1235 [Δ*acdo1*] remained incomplete (Figures 7A–C). In medium supplemented with 0.25% protocatechuic acid + 0.5% glucose, control and G1212/YIC102-AYNI1-ACDO1-6H strains achieved complete consumption of protocatechuic acid not before 72 h of cultivation (Figures 7D,E). Under the same conditions, deletion strain G1235 [Δ*acdo1*] started protocatechuic acid consumption after 24 h of cultivation reaching complete depletion at the end of the cultivation period (Figure 7F). The degradation products identified during cultivation differed for all strains (Supplementary Figure 9). In the control and overexpressing strain G1212/YIC102-AYNI1-ACDO1-6H, catechol and *cis,cis*-muconic acid were detected. In the control strain grown on medium containing 0.25% protocatechuic acid, catechol reached a maximum concentration of ∼0.27 g/L after 24 h of cultivation while *cis,cis*-muconic acid reached a maximum concentration of ∼0.3 g/L after 48 h (Figure 7A). When the control strain was grown on 0.25% protocatechuic acid + 0.5% glucose, maximum values for catechol (∼0.23 g/L) were reached after 48 h of cultivation, whereas *cis,cis*-muconic acid reached ∼0.36 g/L after 72 h of cultivation (Figure 7D). For the overexpression strain grown on medium supplemented with 0.25% protocatechuic acid, the maximum values were 0.09 g/L catechol after 24 h and 0.35 g/L *cis,cis*-muconic acid after 48 h (Figure 7B). In the presence of 0.25% protocatechuic acid and 0.5% glucose, the maximum results were 0.07 g/L catechol after 48 h and 0.45 g/L *cis,cis*-muconic acid after 72 h (Figure 7E). During cultivation of the deletion strain G1235 [Δ*acdo1*] in the presence of 0.25% protocatechuic acid, hydroxyquinol (maximum 0.24 g/L after 24 h) and catechol (maximum 0.185 g/L after 48 h) were detected. Notably, 0.9 g/L of the substrate protocatechuic acid was still detectable in the medium even after 96 h (Figure 7C). When the same strain was cultivated on medium supplemented with 0.25% protocatechuic acid + 0.5% glucose, catechol (maximum 0.28 g/L after 72 h) and hydroxyquinol (maximum 0.09 g/L after 24 h) were identified (Figure 7F).

**FIGURE 8:**
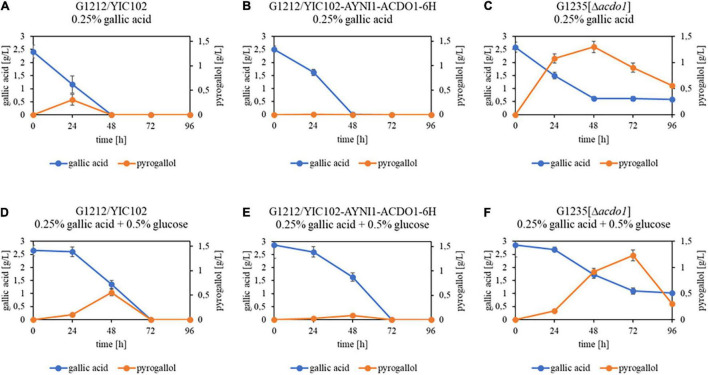
GC-MS analysis of substrate – product conversion during cultivation of *B. raffinosifermentans* on gallic acid. **(A,D)** *B. raffinosifermentans* G1212/YIC102; **(B,E)** *B. raffinosifermentans* G1212/YIC102-AYNI1-ACDO1-6H; **(C,F)** *B. raffinosifermentans* G1235 [Δ*acdo1*]. Strains were cultivated on YMM-NaNO_3_ supplemented with 0.25% gallic acid **(A–C)** or with 0.25% gallic acid plus 0.5% glucose **(D–F)**. Error bars represent one standard deviation.

In an additional experiment, *B. raffinosifermentans* strains G1212/YIC102, G1212/YIC102-AYNI1-ACDO1-6H and G1235 [Δ*acdo1*] were cultivated on HMM-NaNO_3_ supplemented with either 0.25% gallic acid or with 0.25% gallic acid + 0.5% glucose. When gallic acid was the sole source of carbon, the control and G1212/YIC102-AYNI1-ACDO1-6H strain achieved complete degradation of gallic acid within 48 h and within 72 h when glucose was added (Figures 8A,B,D,E). Similar to the experiments using protocatechuic acid, the deletion mutant G1235 [Δ*acdo1*] failed to completely degrade gallic acid under both culture conditions (Figures 8C,F). For the control strain grown in gallic acid-containing medium without and with 0.5% glucose, ∼0.3 g/L and ∼0.55 g/L, respectively, of the gallic acid breakdown (decarboxylation) product pyrogallol was detected (Figures 8A,D). In contrast, only trace amounts of pyrogallol were detected in cultures of the overexpressing strain G1212/YIC102-AYNI1-ACDO1-6H (Figures 8B,E). Finally, the highest pyrogallol concentrations at over 1.2 g/L in both cultivation conditions were measured in the deletion strain (Figures 8C,F).

## Discussion

*Blastobotrys raffinosifermentans* is a relatively recently discovered non-conventional, non-pathogenic, imperfect, haploid yeast species, belonging to the subphylum *Saccharomycotina*. An interesting feature of *B. raffinosifermentans* is the presence of many metabolic pathways, including the degradation of *n*-butanol, purines, and tannins (Bischoff et al., 2017). The present study is dedicated to the investigation of catechol-1,2-dioxygenase and its function in the catabolism of aromatic compounds in *B. raffinosifermentans*.

Ring fission by oxygenases is considered to be the main strategy in the degradation of aromatic structures (Fetzner, 2012; Hu and Wu, 2012; Zhang et al., 2012). Depending on the exact position of cleavage, two groups of dioxygenases are distinguished: intradiol and extradiol dioxygenases. In the *B. raffinosifermentans* genome, a single gene could be annotated as catechol-1,2-dioxygenase, belonging to the family of intradiol oxygenases. Protein sequence alignment to a catechol-1,2-dioxygenase from *Candida albicans* (P86029) revealed 34.5% identity. Further alignments of Acdo1p and known bacterial and fungal intradiol ring cleavage dioxygenases are given in Figure 1 and Supplementary Figure 10. The phylogenetic tree in Figure 2 shows that Acdo1p is evolutionarily most closely related to two enzymes of *Aspergillus niger*, whereas representatives of *Aspergillus nidulans*, of the *Candida* clade and enzymes of bacterial origin are more distant.

Enzymes involved in the catabolism of gallic acid and its derivatives, including most reaction products, are considered to be encoded by inducible genes. By ensuring that enzymes are only produced when substrates are present, microorganisms optimize their energy consumption. When *B. raffinosifermentans* control strain G1212/YIC102 and overexpression strain G1212/YIC102-AYNI1-ACDO1-6H were cultivated on YMM-glucose-NaNO_3_, catechol-1,2-dioxygenase activity was only detected in G1212/YIC102-AYNI1-ACDO1-6H (Figures 6A,D), which has non-native induction of this enzyme. This finding indicates that Acdo1p, similar to the gallic acid decarboxylating enzyme Agdc1p (Meier et al., 2017), is inducible, which was subsequently confirmed by a nested qRT-PCR assay (Figure 4). Similar to *AGDC1*, the *ACDO1* gene was also found to be induced by the presence of gallic acid and protocatechuic acid, the direct precursors of pyrogallol and catechol, respectively, in the tannic acid metabolic pathway of *B. raffinosifermentans*. Moreover, for both genes *AGDC1* and *ACDO1*, highest relative induction levels were observed after 8 h cultivation (Figure 4). The relative expression level of *ACDO1* for protocatechuic acid was approximately 86% lower than that for gallic acid. The analysis of *AGDC1* and *ACDO1* promotors exhibited that they share no identical sequences (see Results Section “Expression Analysis of *ACDO1* on Various Carbon Sources”). It is therefore possible that they are activated differently by specific regulatory elements. Glucose and hydroxylated aromatic acids other than protocatechuic acid and gallic acid that were tested in this study did not show any positive effect on *ACDO1* expression (Figure 4). A similar result was also found for *B. raffinosifermentans* gallic acid decarboxylase, where non-substrate substances had no effect on gene expression (Meier et al., 2017). Induced expression by the presence of enzyme substrates seems to be a common feature of many recently identified genes encoding catechol-1,2-dioxygenases (McFall et al., 1998; Suzuki et al., 2002; Wojcieszynska et al., 2011). However, in other fungal species, catechol-1,2-dioxygenase can also be induced by substrates other than catechol. Martins et al. (2015) showed that in *Aspergillus nidulans*, salicylic acid (2-hydroxybenzoic acid) and benzoic acid also led to the induction of putative catechol-1,2-dioxygenase genes (e.g., AN4532). They confirmed catechol formation from salicylate either directly using decarboxylating salicylate 1-monooxygenase (AN2114) or in a two-step reaction, first converting salicylate to the intermediate 2,3-dihydroxybenzoate by a phenol 2-monooxygenase (AN7418), followed by a non-oxidative decarboxylation step using 2,3-dihydroxybenzoate carboxylase (AN6723, EC 4.1.1.46). The resulting catechol is then rapidly converted to *cis,cis*-muconic acid.

In *A. niger*, Lubbers et al. (2021) identified a salicylate hydroxylase (ShyA) in the salicylic acid metabolic pathway that is able to directly convert salicylate to catechol, hence inducing downstream catechol-1,2-dioxygenase (CrcA) activity. Alternatively, catechol production from the intermediate 2,3-dihydroxybenzoate by means of DhbA was also suggested. Our gene expression data show that 2,3-dihydroxybenzoate does not induce catechol-1,2-dioxygenase activity in *B. raffinosifermentans* (Figure 4), which does not support non-oxidative decarboxylation of 2,3-dihydroxybenzoic acid by a not yet identified 2,3-dihydroxybenzoic acid decarboxylase. On the other hand, the induction of catechol-1,2-dioxygenase by salicylic acid after conversion to catechol by a salicylate hydroxylase is a possible pathway, however, salicylic acid was not tested as a substrate in our study.

The activity of enzymes that degrade aromatic compounds could also be controlled by a higher-level mechanism. Arentshorst et al. (2021) recently identified a TanR/TanX transcriptional activator-repressor module in *A. niger* that is required to control degradation of tannic acid by inducing the expression of tannases and the utilization of gallic acid. A model was suggested, where binding of an inducer to the putative repressor protein would lead to the dissociation of the repressor from the transcription factor/repressor complex, hence activating the transcription factor. However, the inducer in case of the gallic acid utilization pathway is still unknown. Interestingly, the deletion of the gene encoding the putative repressor protein TanX led to the constitutive expression of at least two tannase degrading enzymes, which can be useful when trying to biotechnologically produce aromatic-degrading enzymes without having to rely on the presence of a specific inducer, especially since overexpression of the transcription factor TanR could also lead to constitutive gene expression.

The *in vitro* analysis of *B. raffinosifermentans* catechol-1,2-dioxygenase properties was based on the construction of the *B. raffinosifermentans* strain G1212/YIC102-AYNI1-ACDO1-6H, which allowed expression of recombinant protein with a C-terminal 6His-tag (Acdo1-6hp). The enzyme was successfully purified and characterized (Table 1). SDS-PAGE and Western Blot analysis confirmed a predicted molecular mass of 37.76 kDa for the denatured protein (Supplementary Figure 4A). Size exclusion chromatography of the native protein showed a molecular mass of 79.7 kDa, thus indicating that Acdo1-6hp is a dimeric protein, similar to most known catechol-1,2-dioxygenases (Briganti et al., 1997; Strachan et al., 1998; Potrawfke et al., 2001; Tsai and Li, 2007). Also similar to other catechol-1,2-dioxygenases, the optimum pH for *B. raffinosifermentans* catechol-1,2-dioxygenase is between pH 7.0 – 8.0 with the highest activity measured at pH 7.5 (Briganti et al., 1997; Wang et al., 2001; Camargo et al., 2012; Guzik et al., 2013; Supplementary Figure 4B). Acdo1-6hp is stable (more than 85% of maximum activity) between 4 and 30°C for at least 23 h. An increase of the temperature to 40°C led to a decrease of the enzyme activity to 85% after 7 h and to complete loss of activity after 23 h. At 50°C, the enzyme activity dropped to 60% within 1 h and was completely absent after 7 h of incubation (Supplementary Figure 5). A catechol-1,2-dioxygenase described by Tsai and Li (2007) in *Candida albicans* TL3 was also found to be stable for 30 min up to 40°C, however, longer incubation times were not assayed. Long et al. (2016) described a catechol-1,2-dioxygenase from *Candida tropicalis* JH8 and observed less than 85% activity of the enzyme after only 1 h at 30°C and only residual activity of about 20% after 20 h, whereas Acdo1p shows still more than 85% of maximum activity.

Acdo1-6hp is also more stable compared to recently described bacterial enzymes. Incubating the enzyme at up to 35°C for 3 h kept catechol-1,2-dioxygenase from *Paracoccus* sp. (Aravind et al., 2021) stable, but at 40°C, relative activity dropped to 60%. Guzik et al. (2013) characterized a catechol-1,2-dioxygenase from *Stenotrophomonas maltophilia* strain KB2 with maximum activity at 40°C, but found this enzyme to be not very stable at this temperature (with a half-life at 40°C of 3 h). These results indicate that thermal stability of Acdo1p can be superior to many other known bacterial and fungal intradiol dioxygenases, especially when longer incubation processes at 40°C are required. Purified Acdo1-6hp, stored at 4°C in PBS buffer pH 7.4, retains 99% activity after 55 days. In contrast, stored as crude extract at 4°C, the enzyme loses 60% of initial activity within 2 days. A similar behavior was reported for catechol-1,2-dioxygenases from *Acinetobacter radioresistens* (Briganti et al., 1997) and *Rhodococcus rhodochrous* NCIMB 13259 (Strachan et al., 1998). Freezing of the enzyme, however, results in complete loss of activity.

A number of potential cofactors, mainly metal ions, were tested for their ability to influence Acdo1-6hp activity (Table 2). The strongest inhibiting effect was exerted by AlCl_3_ and ZnSO_4_. However, also the commercial protease inhibitor cocktail (cOmplete™ EDTA-free Protease Inhibitor Cocktail) initially used during the purification procedure almost completely abolished enzyme activity. This is a reminder that protease inhibitors used during enzyme purification have to be selected with care. Addition of iron salts to the reaction mixture also had a negative impact on Acdo1-6hp activity. In general, sulfate salts, with the exception of manganese-sulfate, decreased the activity of catechol-1,2-dioxygenase more than chloride salts.

Of the hydroxylated aromatic compounds used in a substrate specificity test (Table 4), the expected products of catechol-1,2-dioxygenase activity were only found for catechol, pyrogallol and hydroxyquinol. Degradation of catechol leads to the production of *cis,cis*-muconic acid, which indicates that, as expected for a catechol-1,2-dioxygenase, Acdo1-6hp is an intradiol dioxygenase and is not able to cleave the aromatic ring in position 2,3, where the formation of 2-hydroxymuconate semialdehyde would be expected. The kinetic parameters of the *in vitro* degradation of catechol, pyrogallol and hydroxyquinol by catechol-1,2-dioxygenase (Table 3) revealed the highest affinity for catechol (*K*_M_ = 4.0 ± 1.0 μM) compared with hydroxyquinol (*K*_*M*_ = 8.1 ± 1.9 μM) and pyrogallol (*K*_M_ = 101 ± 19 μM). Aravind et al. (2021) had presented a comprehensive comparison of *K*_M_ values of different bacterial catechol-1,2-dioxygenases. Their listing showed values that varied from 1.1 μM for *Rhodococcus rhodochrous* or 1.4 μM for *Rhodococcus opacus* to 85.19 μM in *Pseudomonas putida*. Beyond this comparison, other catechol-1,2-dioxygenases with substantially higher substrate affinity were identified in *Alcaligenes eutrophus* and *Pseudomonas* sp. strain MT1 (Sauret-Ignazi et al., 1996; Cámara et al., 2007). And although the catalytic efficiency of the enzyme in *B. raffinosifermentans* (*k*_cat_/*K*_M_ = 3.9 μM^–1^ s^–1^) is higher than that of *Rhodococcus opacus* 1CP and of one dioxygenase in *Pseudomonas sp*. strain MT1 (C12O_salD_) (3.4 and 1 μM^–1^ s^–1^, respectively), a variety of other bacterial enzymes show substantially greater *k*_cat_/*K*_M_ values (Table 5).

**TABLE 5:** Kinetic parameters for catechol-1,2-dioxygenases of bacterial and fungal origin toward catechol.

Source	*K*_M_ (μM)	*k*_cat_ (s^–1^)	*k*_cat_/*K*_M_ (μM^–1^ s^–1^)	References
*B. raffinosifermentans* (Acdo1p)	4.0	15.6	3.9	This study
*Acinetobacter radioresistens* LMG S13 (CatA)	2.04	24.2	11.8	Micalella et al., 2011
*Alcaligenes eutrophus* CH34 (CatA)	0.25	20	67	Sauret-Ignazi et al., 1996
*Rhodococcus rhodochrous* (CatA)	1.1	11.6	9.7	Strachan et al., 1998
*Pseudomonas* sp. strain MT1 (C12O*_catA_*)	0.8	8.7	10.9	Cámara et al., 2007
*Pseudomonas* sp. strain MT1 (C12O*_salD_*)	7.5	7.5	1	Cámara et al., 2007
*Rhodococcus opacus* 1CP (CatA)	3	10.2	3.4	Matera et al., 2010
*Aspergillus niger* UBC 814	52	-	-	Ninnekar and Vaidyanathan, 1981
*Candida tropicalis* JH8 (Cato)	9.2	-	-	Long et al., 2016
*Candida albicans* TL3 (HQD2)	9.3	28	3.0	Tsai and Li, 2007
*Trichosporon cutaneum* WY 2-2	9.0	-	-	Itoh, 1981
*A. niger* NRRL3_02644 (HqdA)	7.6	0.32	0.04	Semana and Powlowski, 2019
*A. niger* NRRL3_04787 (CrcA)	2.4	8.96	3.67	Semana and Powlowski, 2019
*A. niger* NRRL3_05330	20.5	0.44	0.021	Semana and Powlowski, 2019

In contrast, reliable data on kinetic parameters of fungal catechol-1,2-dioxygenases are much less available. Ninnekar and Vaidyanathan (1981) had characterized the corresponding enzyme in *Aspergillus niger* and found a *K*_*M*_ value of 52 μM. In the same year, Itoh (1981) determined a *K*_*M*_ of 9.0 μM in the yeast *Trichosporon cutaneum* WY 2-2. At a similar level, lagging behind the affinity of Acdo1p, are the values of *Candida tropicalis* JH8 and *Candida albicans* TM3 (9.2 and 9.3 μM, respectively) (Tsai and Li, 2007; Long et al., 2016). For *C. albicans*, the catalytic efficiency *k*_*cat*_/*K*_*M*_ is given as 3.0 μM^–1^ s^–1^, which is also exceeded by Acdo1p. Last but not least, the *k*_*cat*_/*K*_*M*_ values of three recently discovered intradiol ring cleavage dioxygenases in *A. niger* are also less than those of Acdo1p, which thus appears to be superior to other catechol-1,2-dioxygenases of fungal origin in terms of both affinity and catalytic efficiency.

To investigate the *in vivo* role of Acdo1p in tannin catabolism by *B. raffinosifermentans*, a deletion mutant strain, G1235 [Δ*acdo1*] as well as an overexpressing *ACDO1* strain (G1212/YIC102-AYNI1-ACDO1-6H) were constructed. The deletion mutant G1235 [Δ*acdo1*], which did not grow on medium supplemented with only gallic acid or protocatechuic acid as the sole source of carbon, showed growth for 24 h when 0.5% glucose was added (Figures 5C,F). This confirms the importance of Acdo1p in the catabolism of at least these two hydroxylated aromatic acids. GC-MS analysis of the supernatant revealed that when gallic acid was the sole source of carbon, it was completely metabolized by the control and overexpression strain G1212/YIC102-AYNI1-ACDO1-6H within 48 h of cultivation, whereas incomplete metabolism was measured for the deletion mutant (Figure 8). Supplementing glucose as an additional carbon source delayed the degradation of gallic acid by about 24 h. In the spent medium of control and deletion mutant strains, the only gallic acid degradation product detected was pyrogallol. Further degradation of pyrogallol by G1235 [Δ*acdo1*] was very slow, thus pyrogallol could be detected throughout the whole cultivation process (Figures 8C,F). The culture medium of overexpression strain G1212/YIC102-AYNI1-ACDO1-6H contained only trace amounts of pyrogallol, indicating a significantly accelerated degradation process (Figures 8B,E). *In vitro* analysis confirmed the affinity of Acdo1-6hp for pyrogallol (Table 3), and nested quantitative RT-PCR as well as microarray analysis confirmed the induction of *B. raffinosifermentans* catechol-1,2-dioxygenase by gallic acid (Figure 4; Meier et al., 2017, GEO accession GSE195801). When Sietmann et al. (2010) investigated pyrogallol transformation by wild-type *B. raffinosifermentans* by GC-MS analysis, they detected a product showing similarities with *cis,cis*-muconic acid. They proposed 2-hydroxymuconic acid as a transformation product, which fits our *in vitro* enzyme analysis of purified Acdo1-6hp (Table 4). Therefore, although pyrogallol degradation products were not identified in our study, 2-hydroxymuconic acid remains the most likely product. However, an enzyme responsible for the further degradation of 2-hydroxymuconic acid has not been identified. Also, a possible spontaneous isomerization of 2-hydroxymuconic acid into oxalocrotonate (Subramanya et al., 1996) creates uncertainty in the proposed tannic acid degradation pathway (Supplementary Figure 7). Further investigations will be indispensable to identify these so-far elusive degradation products.

*Blastobotrys raffinosifermentans* gallic acid decarboxylase is involved in the decarboxylation of both gallic acid and protocatechuic acid (Meier et al., 2017). Investigations of the *in vivo* role of gallic acid decarboxylase (Agdc1p) in *B. raffinosifermentans* had revealed that removal of the carboxylic group is not the only path for the degradation of protocatechuic acid (Meier et al., 2017). The spent culture media of the strains G1212/YIC102, G1212/YIC102-AYNI1-ACDO1-6H and G1235 [Δ*acdo1*] grown on HMM-NaNO_3_ supplemented with 0.25% protocatechuic acid or 0.25% protocatechuic acid and 0.5% glucose were therefore screened by GC-MS analysis for the presence of possible protocatechuic acid degradation products. The only reaction products detected during cultivation of the control strain G1212/YIC102 and the overexpression strain G1212/YIC102-AYNI1-ACDO1-6H were catechol and *cis,cis*-muconic acid (Figures 7A,B,D,E). However, the rate at which the products were formed and their maximum concentrations measured in the medium depended strongly on the genotype and medium used. In the cultures in which protocatechuic acid served as the sole substrate, catechol was measured after only 24 h in both the control strain and the overexpressing strain, indicating rapid utilization of the substrate (Figures 7A,B). However, in the *ACDO1* overexpressing strain, the maximum measured catechol concentration was much lower than in the wild-type strain, indicating that the overexpressed catechol-1,2-dioxygenase converted the newly formed catechol to *cis,cis*-muconic acid more rapidly than the wild-type strain. Accordingly, the rate of *cis,cis*-muconic acid formation also differs. Whereas the wild type only completely converted catechol after 48 h, in the overexpression strain, the maximum *cis,cis*-muconic acid concentration was observed after 24 h. The fact that the product concentration remains constant for a longer period thereafter also indicates that, as expected, catechol-1,2-dioxygenase overexpression has no effect on downstream degradation processes, which could be confirmed by a longer observation time beyond 96 h. The delayed catechol formation in the medium with protocatechuic acid and added glucose (Figures 7D,E) suggests that glucose is first used as a carbon source before the degradation of protocatechuic acid takes place. But again, the downstream step of catechol degradation occurred much faster in the overexpression strain.

In contrast, the G1235 [Δ*acdo1*] deletion mutant showed a different pattern, and degradation of protocatechuic acid was incomplete when this served as the sole source of carbon. In the supernatants, the products catechol and hydroxyquinol were found after 24 h (Figure 7C). Although the initial catechol formation resembled that in the control strain, there was almost no conversion to *cis,cis*-muconic acid. This can be seen as an indication that the deleted catechol-1,2-dioxygenase is not substituted by another enzyme. However, glucose as an additional carbon source leads to complete protocatechuic acid degradation (Figure 7F).

These results indicate that degradation of protocatechuic acid through catechol in *B. raffinosifermentans* is the preferred but not the only metabolic route. The normal microbial strategy of direct ring cleavage of protocatechuic acid is unlikely, which is supported by the fact, that when protocatechuate 3,4-dioxygenase PrcA of *A. niger* (A2R1P9.1, Lubbers et al., 2019) was compared with the *B. raffinosifermentans* genome using BlastP in the GRYC yeast database (see footnote 5), the best hit was to Acdo1p (ARAD1D18458g) with 33.2% identity. However, since we had previously shown that Acdo1p does not accept protocatechuic acid as substrate (Table 4), ring cleavage by this or another enzyme with even less sequence identity to PrcA seems very unlikely.

Hydroxyquinol has also been detected in the supernatant of *B. raffinosifermentans* LS3 cultures (Sietmann et al., 2010), indicating a simultaneous oxidative and non-oxidative decarboxylation of protocatechuic acid. The oxidative decarboxylation of protocatechuic acid through hydroxyquinol was already described for the pathogenic yeasts *Candida parapsilosis* and *Rhodotorula rubra* (Eppink et al., 1997; Holesova et al., 2011) and has also recently been shown in the filamentous fungus *A. niger*, where a gene annotated as protocatechuate hydroxylase (*phyA*) was identified using whole-genome transcriptome data (Lubbers and de Vries, 2021). Our GC-MS analysis of supernatant metabolites showed only trace amounts (∼0.01 g/L) of hydroxyquinol in the case of the *B. raffinosifermentans* control strain (Figure 7D). Microarray data analysis pointed to a number of potential 4-hydroxybenzoate 1-hydroxylase candidate enzymes converting protocatechuic acid into hydroxyquinol (ARAD1C03498g, ARAD1D18392g, ARAD1D27984g, ARAD1C08580g, ARAD1C00330g, and ARAD1C08558g), based on gene annotations, descriptions and Gene Ontology terms (see Results Section “Expression Analysis of *ACDO1* on Various Carbon Sources” and Supplementary Figure 8). In order to gain more information on these genes, a BlastP search of the above mentioned protocatechuate hydroxylase (PhyA) against the *B. raffinosifermentans* genome was performed and resulted in four hits of more than 30 percent identity. One of them, ARAD1A17402g, is described in our genome data to exert monooxygenase or oxidoreductase activity. Three genes, however, had already been candidates from our microarray data: ARAD1C08558g (35.9% identity), ARAD1D18392g (34.3% identity), and the gene with the strongest match, ARAD1D27984g (51.8% identity), the last one being designated as salicylate hydroxylase (Supplementary Table 5). Future *in vivo* studies must show whether one of these genes represent functional 4-hydroxybenzoate 1-hydroxylases in *B. raffinosifermentans*.

**FIGURE 9:**
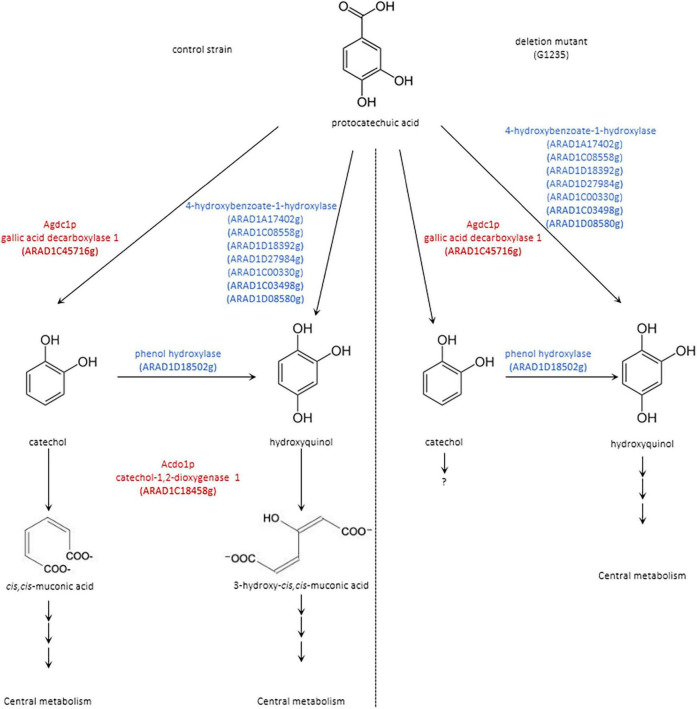
Proposed pathway for the degradation of protocatechuic acid in *B. raffinosifermentans* **(left)** control strain and **(right)** deletion mutant. Scheme shows compounds detected by GC-MS analysis in black; proposed enzymes involved in putative reactions are shown in blue. Agdc1p = gallic acid decarboxylase and Acdo1p = catechol-1,2-dioxygenase are highlighted in red.

Another gene annotated as phenol hydroxylase, ARAD1D18502g, is probably also involved in this degradation pathway and catalyzes the conversion of catechol into hydroxyquinol. Interestingly, a sequence alignment showed 29.2% identity with PhhA from *A. niger* and 46.5% identity with Mnx3 from *C. parapsilosis* (Supplementary Table 5). A proposed pathway for the degradation of protocatechuic acid by *B. raffinosifermentans* is shown in Figure 9.

In this study, physiological and transcriptome analysis was used to unravel the catabolism of hydroxylated aromatic acids in *B. raffinosifermentans*. The results confirmed that the genes encoding enzymes involved in the catabolic pathway of gallic acid and protocatechuic acid are inducible and that aromatic acid substrates are the inducers. Our previous study had shown that the construction of gallic acid decarboxylase disruption mutants proved gallic acid decarboxylase (Agdc1p) to be the only enzyme responsible for the transformation of gallic acid (Meier et al., 2017). In contrast to this, this new study using catechol-1,2-dioxygenase disruption mutants showed that, in the absence of Acdo1p, an alternative pathway takes over the degradation of protocatechuic acid. Both the substrate specificity and the ability for long-term storage without significant loss of activity make *B. raffinosifermentans* catechol-1,2-dioxygenase (Acdo1p) a promising enzyme candidate for commercial use. In particular, the enzyme could be used in a number of industrial applications, such as bioremediation processes or chemical synthesis. Further optimization of culture conditions as well as strain engineering could also contribute to a more efficient conversion of protocatechuic acid to *cis,cis*-muconic acid. *B. raffinosifermentans* has already proven to be a good alternative host relative to *S. cerevisiae* in the past (Brückner et al., 2018), especially when chemical synthesis is to be combined with bioremediation processes. Catechol-1,2-dioxygenase (Acdo1p) is another example of how the use of *B. raffinosifermentans*’ own enzymes can help to improve the production of industrially relevant chemicals.

## Data Availability Statement

The datasets presented in this study can be found in online repositories. The names of the repository/repositories and accession number(s) can be found below: https://www.ncbi.nlm.nih.gov/geo/, GSE195808.

## Author Contributions

AM carried out the construction of the *E. coli* and *B. raffinosifermentans* strains, enzyme activities determination, and Acdo1p analysis, prepared the quantitative reverse transcriptase PCR analysis. AM, AH, MM, and SW participated in microarray design, hybridization, gene annotation, and gene expression analysis as well as visualization. AM and FM performed the GC–MS analysis. MM and AM prepared the microscopic analysis. RB gave several useful suggestions. AM, FM, and GK drafted the manuscript. All authors read and approved the final manuscript.

## Conflict of Interest

The authors declare that the research was conducted in the absence of any commercial or financial relationships that could be construed as a potential conflict of interest.

## Publisher’s Note

All claims expressed in this article are solely those of the authors and do not necessarily represent those of their affiliated organizations, or those of the publisher, the editors and the reviewers. Any product that may be evaluated in this article, or claim that may be made by its manufacturer, is not guaranteed or endorsed by the publisher.

## Footnotes

1
https://mycocosm.jgi.doe.gov/cgi-bin/browserLoad?db=Arxad1&position=ARAD1D_4:1512070-1513071

2
https://www.uniprot.org/uniprot/A0A060T9I8

3
https://earray.chem.agilent.com

4
https://services.healthtech.dtu.dk/service.php?SignalP-4.1

5
http://gryc.inra.fr/index.php?page=home

## Abbreviations

ACDO1 gene: catechol-1,2-dioxygenase 1 gene from *B. raffinosifermentans*
Acdo1p: catechol-1,2-dioxygenase 1 protein from *B. raffinosifermentans*
AGDC1 gene: gallic acid decarboxylase 1 gene from *B. raffinosifermentans*
Agdc1p: gallic acid decarboxylase 1 protein from *B. raffinosifermentans*
dcw: dry cell weight
YRC: yeast rDNA integrative expression cassette
YIC: yeast integrative expression cassette
YEPD: complex medium
YMM: yeast minimal medium
U: unit
ORF: open reading frame.
