# Supplementary material for: Characterization of Catechol-1,2-Dioxygenase (Acdo1p) From Blastobotrys raffinosifermentans and Investigation of Its Role in the Catabolism of Aromatic Compounds
Source: Front Microbiol. 2022 Jun 3;13:872298. doi: 10.3389/fmicb.2022.872298 (PMC9204233; doi:10.3389/fmicb.2022.872298)
Supplement: Supplementary file 1 [file Data_Sheet_1.PDF]

Supplementary Table 1. List of bacteria and yeast strains used in this study

**Strain**	**Description**	**Company / Reference**
E. coli XL1 blue	[*recA1, endA1, gyrA96, thi-1, hsdR17, supE44, relA1, lac* [F'proABlacl q Z DM15 Tn10 (Tetr)]	Invitrogen<br>(Grand Island, NY, USA)
E. coli DH5α	[F– Φ80d*lac*ZΔM15 Δ(*lac*ZYA-*arg*F) U169, deoR, *rec*A1, *end*A1, *hsd*R17 (r_K–, m_K+) *pho*A *sup*E44, λ–, *thi*-1, *gyr*A96 *rel*A1]	Pierce-Thermo-Fisher Scientific (Rockford, USA)
B. raffinosifermentans LS3	wild-type strain	Kunze and Kunze (1994)
B. raffinosifermentans G1212	auxotrophic mutant strain, [aleu2 atrp1::ALEU2]	Steinborn *et al*. (2007)
B. raffinosifermentans G1212/YIC102	YIC102 control strain, transformed with plasmid Xplor2.2 lacking the *ACDO1* expression module	this study
B. raffinosifermentans G1212/YRC102	YRC102 control strain, transformed with plasmid Xplor2.2 lacking the *ACDO1* expression module	this study
B. raffinosifermentans G1240	G1212/YIC102-AYNI1-ACDO1-6H,<br>*ACDO1* over-expression strain	this study
B. raffinosifermentans G1241	G1212/YRC102-AYNI1-ACDO1-6H,<br>*ACDO1* over-expression strain	this study
B. raffinosifermentans G1235	G1212 [Δ*acdo1*],<br>Δ*acdo1* deletion mutant strain	this study

Supplementary Table 2. List of plasmids used in this study

**Plasmid name**	**Features**	**Company / Reference**
pCR4®4-TOPO®	promoter *lacl*, Amp^r, Kan^r;<br>used for cloning in *E. coli*	Invitrogen (Life Technologies)
pBS-AYNI1-PHO5-SS	inducible *AYNI1* promoter, Amp^r;<br>used for cloning in *E. coli*	Böer *et al.*, 2009c
pB25S-ALEU2-ATRP1m-SS	*TRP1m selection*, Kan^r;<br>used for generation of transformation vector	Böer *et al.*, 2009c
Xplor2.2	control plasmid (lacking the *ACDO1* expression module)	Böer *et al.*, 2009b
YRC102-AYNI1-ACDO1-6H	used for transformation of *B. raffinosifermentans*	this study
YIC102-AYNI1-ACDO1-6H	used for transformation of *B. raffinosifermentans*	this study
pBS-ALEU2-TRP1m	used for generation of Δ*acdo1* deletion module	Steinborn *et al.*, 2007
pBS-ALEU2-TRP1m_ Δ*acdo1*	Δ*acdo1* deletion module	this study

Supplementary Table 3. Primers used for the construction of deletion mutant strain G1235 [Δ*acdo1*].

**Primers for the construction of *B. raffinosifermentans* G1235 [Δ*acdo1*]**	
**Primer**	**Sequence (5‘ → 3‘)**
**1_ovs_ex_forward**	TAATCAACAGAATT TGACTAT TGATTTCCCCACC
**1_ex_reverse**	GGGGAATCAATCGAGAAATAATAAA
**2_ovs_ex_forward**	ATACAGACTCGATCGACGATTCCTTACCTATGTAGG
**2_ex_reverse**	GGGAATTAGCGGCC TGTAATCAATGTAAAGGACCAGG
**3_Marker_forward_ovs**	CTCGATTGATTCCCCTTTCAATCGACGATTGCA
**3_Marker_reverse**	CGATCGAGTCTGTATTGAAG
**ACDO_screen_fwd**	CAGCGTCTTTGCCCCTGATA
**ACDO_screen_rev**	GCCAAGCTGCTTCCAGTTTC

Supplementary Table 4. Primers used for the analysis of *ACDO1* expression levels.

**Primers for nested quantitative RT PCR**		
**Reaction step**	**Primer**	**Sequence (5‘ → 3‘)**
**Step 1**<br>**cDNA synthesis**	(dT) 15V-RTA	TGA CAG GAT ACC ATA CAG ACA CTA TTT TTT TTT TTT TTT V
**Step 2**<br>**First PCR synthesis**	RTA-1 rv	TGA CAG GAT ACC ATA CAG ACA C
	ACDO1-V fw	TTC GAT GCT CAG AGC CGA C
	TFC1-3 fw	TGA AGA AGA GCA CCA AGC A
**Step 3**<br>**Second PCR synthesis**	RTA rv	TGA CAG GAT ACC ATA CAG ACA CTA
	ACDO1-III fw	AAG GTT GTC AAC GGA GTC CC
	TFC1-1 fw	ACA ACA AGA TGA AAA CGC

Supplementary Table 5. Sequence comparison of *B. raffinosifermentans* translated ORFs as depicted in Figure 9 with validated versions from public databases (highlighted in orange). *B. raffinosifermentans* sequences are taken from the GRYC database, the PhyA sequence is taken from Lubbers and de Vries (2021), and the remaining sequences are taken from UniProt.

Protein name	Organism	Gene ID (Gene name)	% AA Identity	# Amino Acids	UniProt Accession
4-hydroxybenzoate 1-hydroxylase	*C. parapsilosis*	CPAR2_102790 (Mnx1)		479	G8B709
	B. raffinosifermentans	ARAD1C00330g	8.8	347	A0A060SYV9
		ARAD1C03498g	13.1	405	A0A060SZ98
		ARAD1D18392g	44.9	426	A0A060TFY0
		ARAD1D27984g	28.0	420	A0A060TH41
		ARAD1C08580g	4.8	85	A0A060SZJ7
		ARAD1C08558g	32.2	414	A0A060T0J4
		ARAD1A17402g	17.9	721	A0A060T3N3
	A. niger	NRRL3_4659 (PhyA)		422	
	B. raffinosifermentans	ARAD1C00330g	12.1	347	A0A060SYV9
		ARAD1C03498g	18.5	405	A0A060SZ98
		ARAD1D18392g	34.3	426	A0A060TFY0
		ARAD1D27984g	51.8	420	A0A060TH41
		ARAD1C08580g	4.5	85	A0A060SZJ7
		ARAD1C08558g	35.9	414	A0A060T0J4
phenol hydroxylase	*A. niger*	NRRL3_08551 (PhhA)		636	A2QGH7
	B. raffinosifermentans	ARAD1D18502g	29.2	686	A0A060TFY3
phenol hydroxylase	*C. parapsilosis*	CPAR2_205970 (Mnx3)		712	G8BGH1
	B. raffinosifermentans	ARAD1D18502g	46.5	686	A0A060TFY3
catechol-1,2-dehydroxylase	*C. albicans*	CAALFM_C402230CA (Hqd2)		303	P86029
	B. raffinosifermentans	ARAD1D18458g	30.2	334	A0A060T9I8
catechol-1,2-dehydroxylase	*A. niger*	NRRL3_02644 (HqdA)		329	A2QAP8
	B. raffinosifermentans	ARAD1D18458g	49.4	334	A0A060T9I8
gallic acid decarboxylase	*N. crassa*	NCU05730		227	Q7SB82
	B. raffinosifermentans	ARAD1C45716g	53.2	231	A0A060TAG5

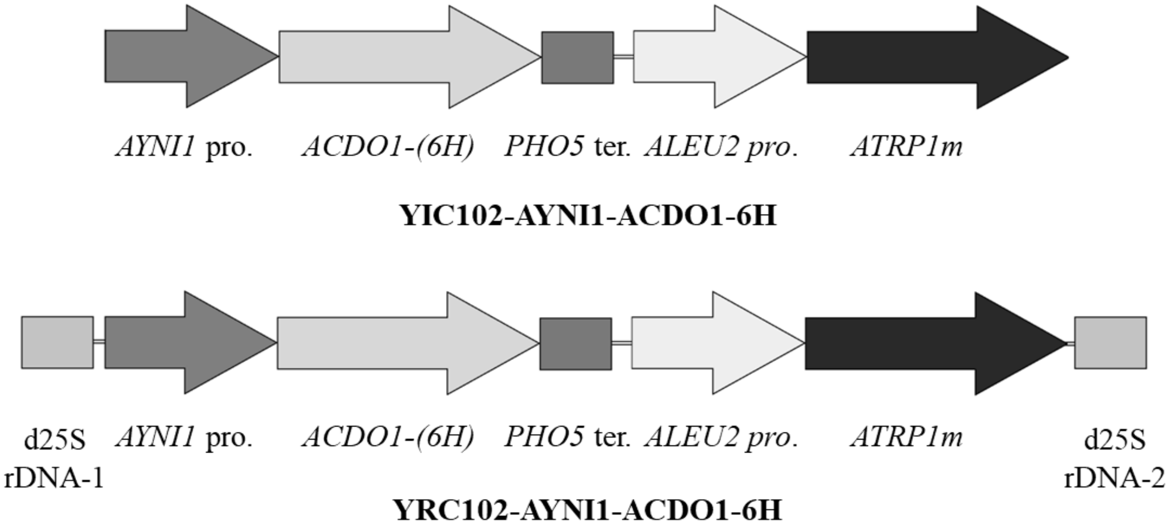

Supplementary Figure 1 Physical maps of the *ACDO1* yeast integrative expression cassettes.

The cassettes contain one copy of the expression module: *AYNI1* promoter-*ACDO1*-6H-*PHO5*-terminator, selection marker *ATRP1m* together with the *ALEU2* promoter. In the YRC102 cassette, the expression module is flanked by two 25S rDNA sequences for homologous recombination.

(A)

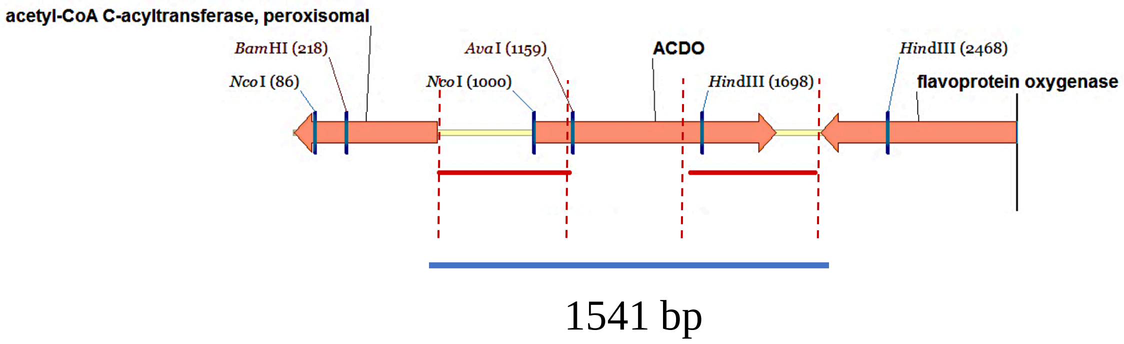

(B)

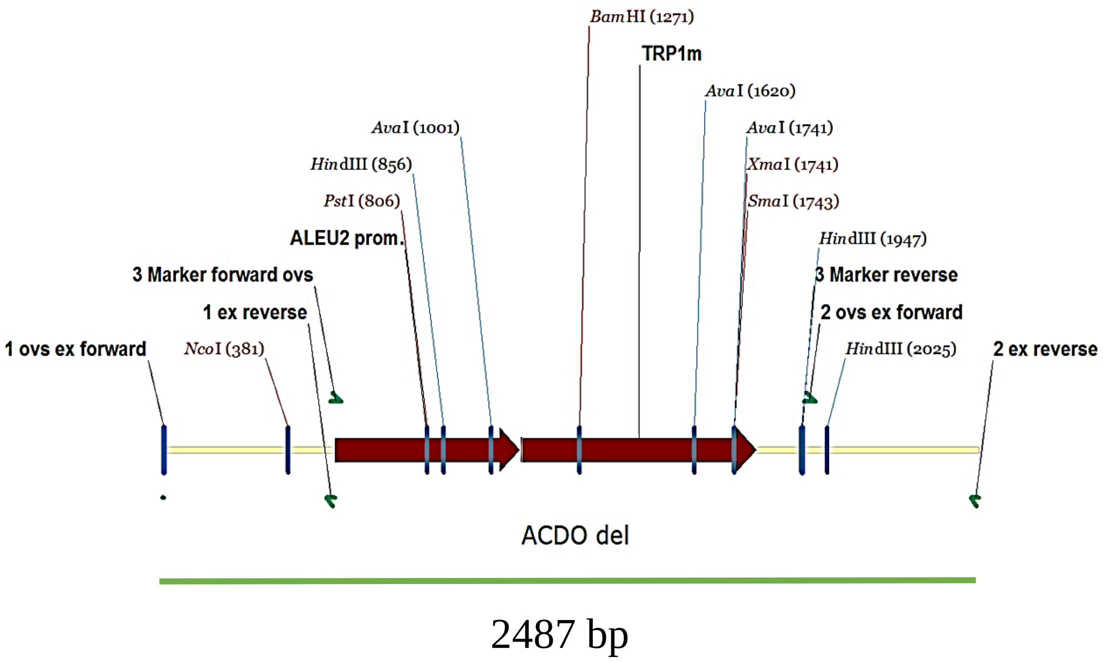

Supplementary Figure 2 Design of *ACDO1* gene deletion cassettes.
(A) DNA fragment used for construction of *ACDO1* deletion cassette. To avoid affecting putative nearby genes, deletion was restricted to a 995 bp fragment of *ACDO1*: left red line = 381 bp upstream homology fragment + 142 bp of the 5' gene fragment; right red line = 158 bp downstream homology fragment + 361 bp of the 3' gene fragment. Blue line = fragments obtained by PCR after unsuccessful deletion of *ACDO1*. **(B)** Deletion mutant cassette. Yellow line = 523 bp upstream and 519 bp downstream homology fragments; red arrows = *ALEU2* promoter; *ATRP1* = tryptophan marker; green line = fragment obtained by PCR after successful deletion of *ACDO1*.

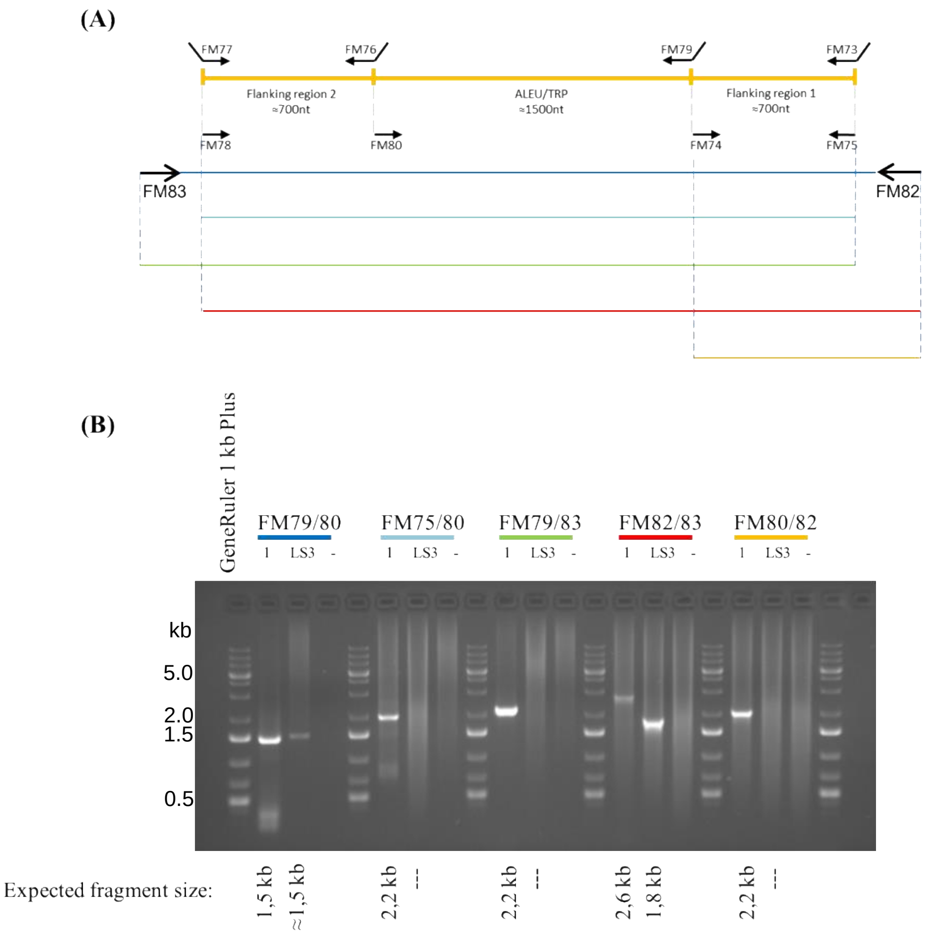

Supplementary Figure 3 Verification of correct integration of *ACDO1* deletion cassettes by PCR.

Integration into the target locus was verified by PCR of isolated genomic DNA. **(A)** Fragments design for verification of correct integration of the deletion cassette into the genome. **(B)** Correct integration yielding a 2487 bp DNA fragment whereas a 1541 bp long fragment represents the unaffected *ACDO1* gene, serving as negative control. 1 – strain number with correct targeted deletion cassette (Δacdo1); LS3 – wild type (control); "–" – no template PCR reaction (control).

(A)

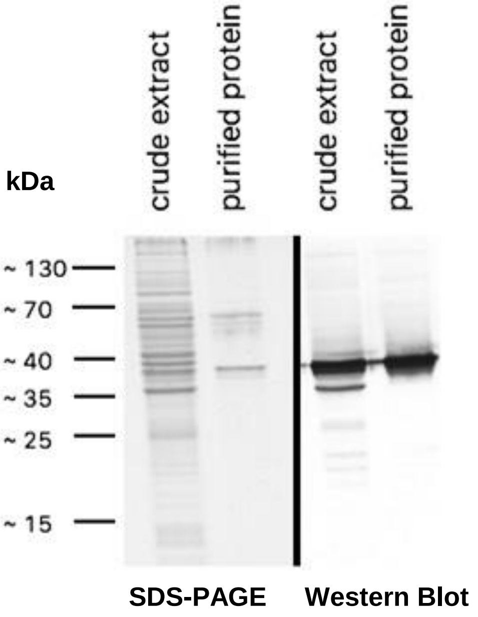

(B)

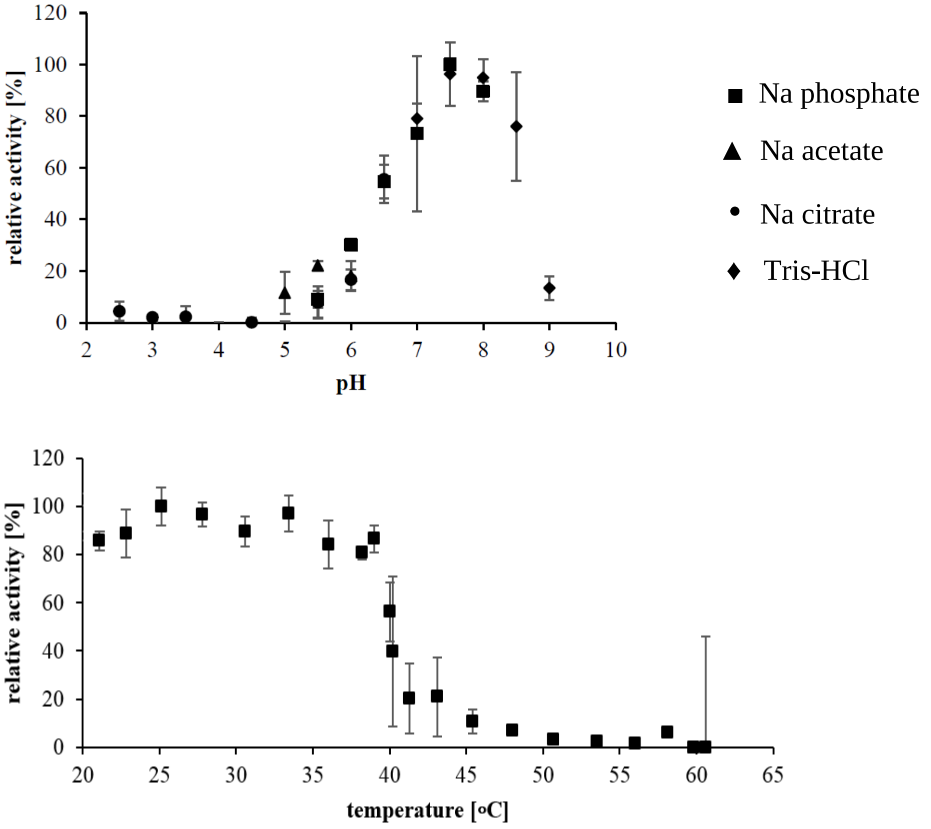

Supplementary Figure 4 Characterization of Acdo1-6hp.
(A) Purification of Acdo1-6hp on Ni Sepharose. SDS-PAGE of crude extract and purified Acdo1-6hp (left). Western blot analysis using anti-polyhistidine antibodies confirms the identity of Acdo1-6hp (right). Protein size values refer to selected fragments of PageRuler^{TM} Plus Prestained Protein Ladder (Thermo Scientific^{TM}) **(B)** Effect of pH (upper panel) and temperature (lower panel) on Acdo1-6hp activity. Maximum enzyme activity was observed at pH 7.5 and 25 °C, respectively.

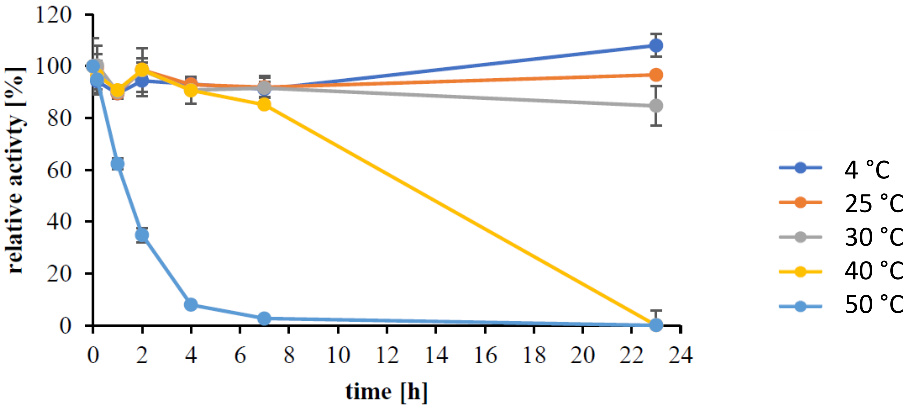

Supplementary Figure 5 Thermal stability of Acdo1p-6hp.
Relative enzyme activity was measured at different temperatures for 23 h at pH 7.5. Measurements were done in triplicate and error bars represent one standard deviation.

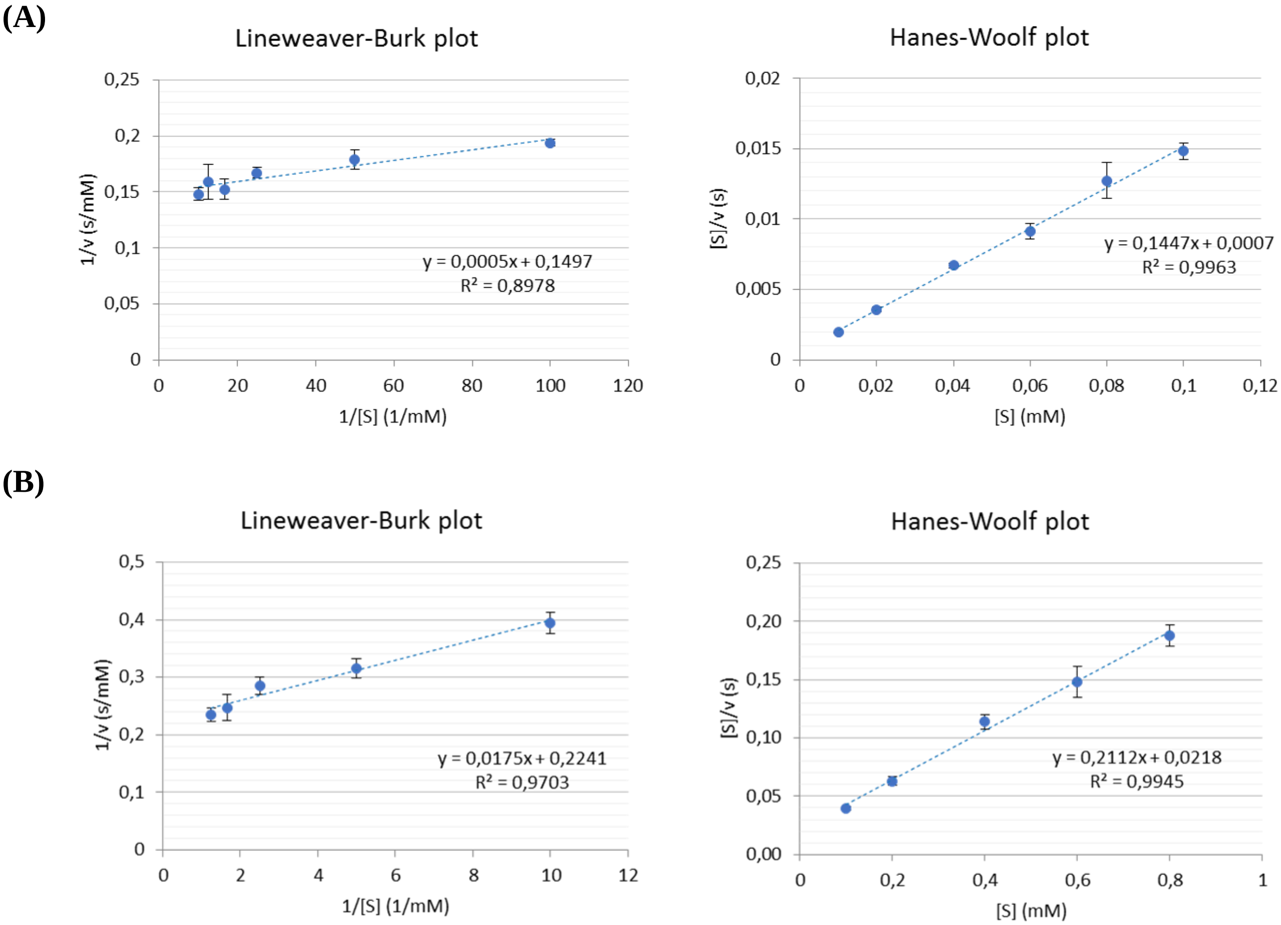

Supplementary Figure 6 Lineweaver-Burk and Hanes-Woolf plots for determination of catechol-1,2-dioxygenase kinetic constants.

(A) Catechol-1,2-dioxygenase activity on catechol, **(B)** catechol-1,2-dioxygenase activity on pyrogallol. Measurements were done in triplicate and error bars represent one standard deviation.

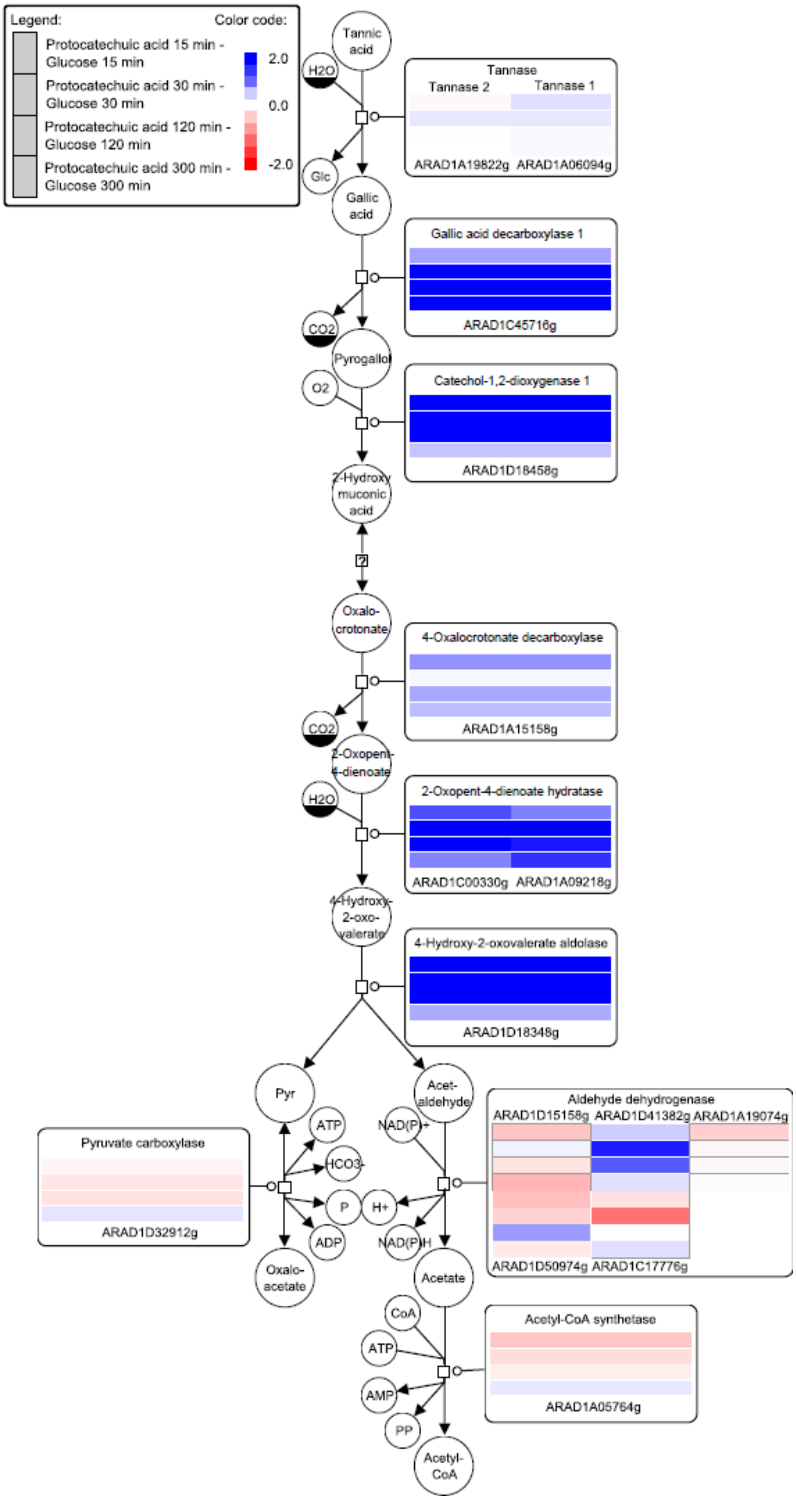

Supplementary Figure 7 Key compounds of the tannin catabolism based on microarray studies of gene regulation by protocatechuic acid.

The Systems Biology Graphical Notation (SBGN) of the metabolic network depicts reactions catalysed by the corresponding enzymes (rectangular square). Enzymes are shaded with color-coded log2 fold change values of time-resolved expression data of the respective genes. The colors represent upregulation (dark blue 2-fold upregulation) and downregulation (dark red 2-fold downregulation) of genes in cells shifted to a medium containing protocatechuic acid as the carbon source compared to cells grown on glucose. Metabolites or enzymes occurring multiple times in the metabolic network are decorated with a clone marker (e.g. NAD+) [graph produced with VANTED – (Junker *et al.*, 2012; Rohn *et al.*, 2012).

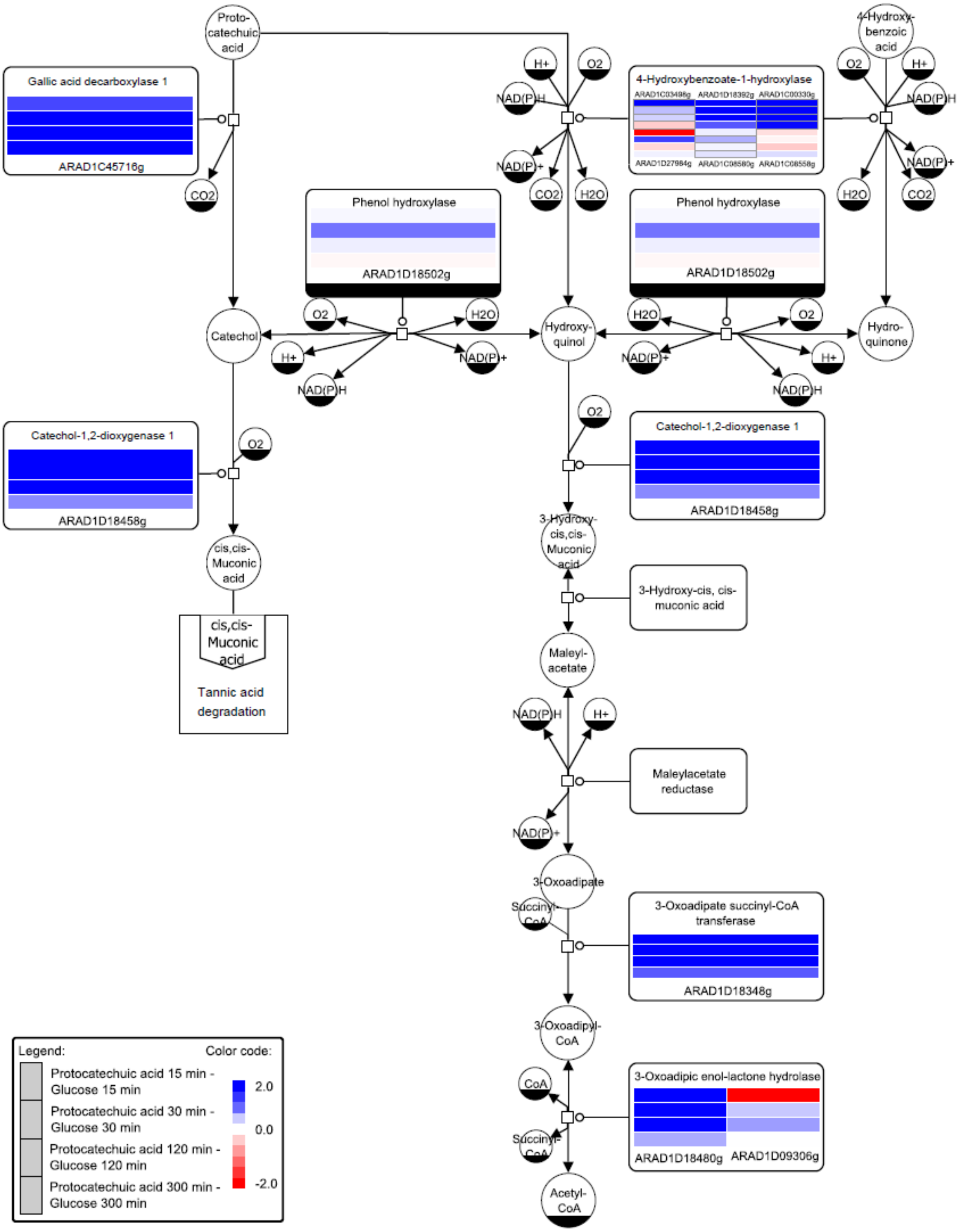

Supplementary Figure 8 Expanded scheme of potential key compounds involved in tannin catabolism based on microarray studies of gene regulation by protocatechuic acid.

The Systems Biology Graphical Notation (SBGN) of the metabolic network depicts reactions catalysed by the corresponding enzymes (rectangular square). Enzymes are shaded with color-coded log2 fold change values of time resolved expression data of the respective genes. The colors represent upregulation (dark blue 2-fold upregulation) and downregulation (dark red 2-fold downregulation) of genes in cells shifted to a medium containing protocatechuic acid as the carbon source compared to cells grown with glucose. Metabolites or enzymes occurring multiple times in the metabolic network are decorated with a clone marker (e. g. NAD+) [graph produced with VANTED – (Junker *et al.*, 2012, Rohn *et al.*, 2012)].

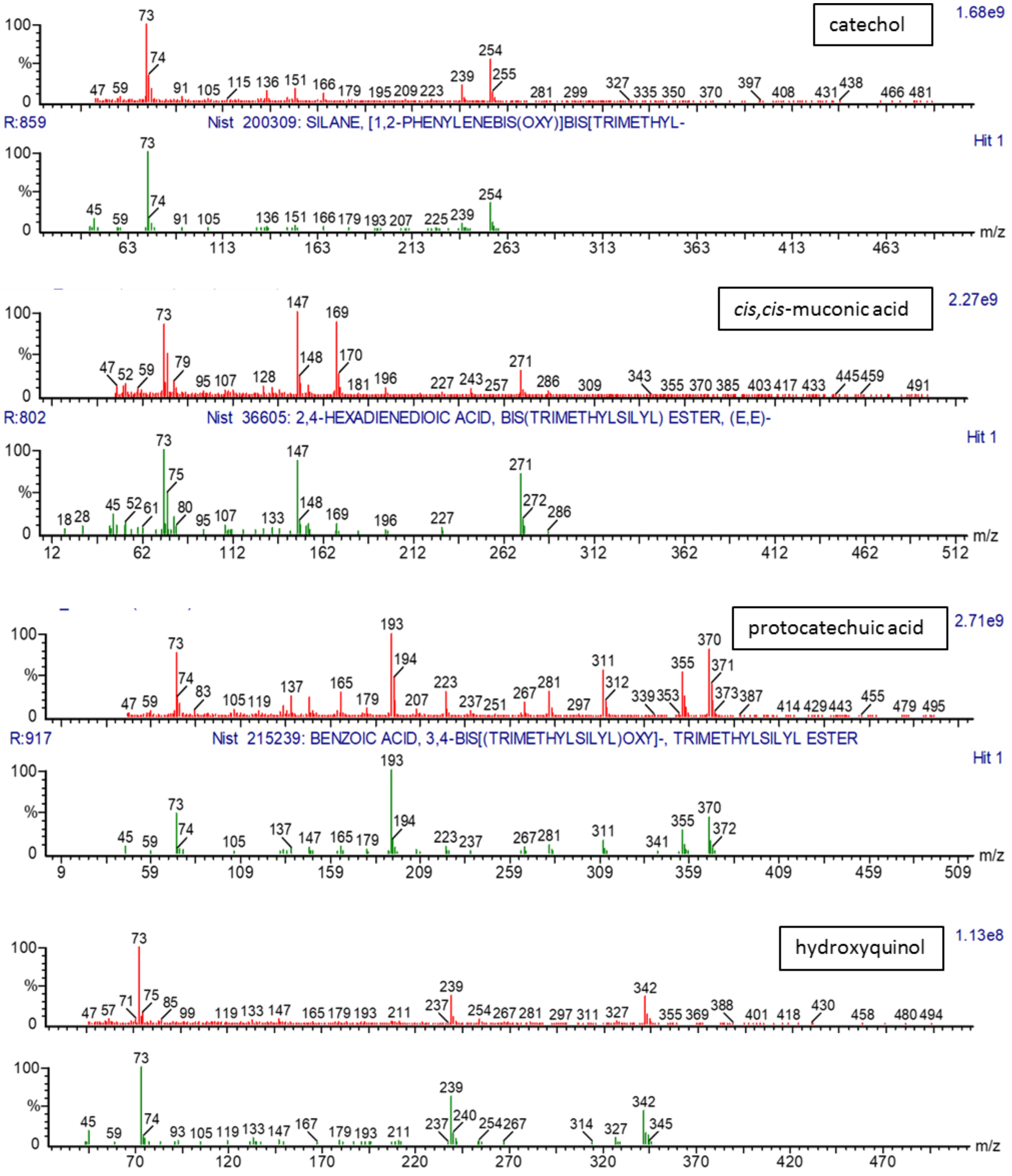

Supplementary Figure 9 GC/MS spectra of identified substances.
Compound spectra (red) were compared with data from a local NIST Mass Spectral Library (green). The best matches were used for identification of catechol, *cis*,*cis*-muconic acid and protocatechuic acid, when probability scores exceeded 0.800. For hydroxyquinol, an internal standard served as a reference because the local version of the NIST library did not allow identification due to lack of a spectrum for silylated 1,2,4-trihydroxybenzene.

```
A0A060T9I8|Acdo1p|B.raffinosiferm.  --------------------------MAGSGLEGYEATSKEDMWDRFFTTQAV-------     27
A2QAP8|HqdA|A.niger                 --------------------------MDPSK---VKIPPMKDLTVDNITENVI-------     24
A2QEL8|NRRL3_5330|A.niger           MSEQQSNGTTPKVTGLSERTRAKWAAASTPEEIAANLPPMLDLTIENITENVM-------     53
A2QNW8|CrcA|A.niger                 ----------------------------------------MPRFDPNFTDNVI-------     13
Q5B4J8|AN4532|A.nidulans            --------------------------------------MASHRFDPKFTDNVV-------     15
Q5AT14|PrcA|A.nidulans              -------------------------------------MSTNPRFDPNFTPYVI-------     16
A2R1P9|PrcA|A.niger                 -------------------------------------MSTNRRFDPNFTPYVV-------     16
A0A0B4ZYX0|Cato|C.tropicalis        -------------------------------------------MSQEFTDAVK-------     10
P86029|Hqd2|C.albicans              -------------------------------------------MSQAFTESVK-------     10
P96984|CatA|R.erythropolis          --------------------------------------------MTDIDTAVATAHASGN     16
P95607|CatA|R.opacus                --------------------------------------------------------GSGS     4
Q43984|CatA|A.guillouiae            -------------------------------------MMMNRQQIDSLVQQMNV------     17
O33950|CatA2|A.lwoffii              ---------------------------------------MNKQAIDALLQKIND------     15
A7LBQ4|CatA|P.putida                -------------------------------------------PMFSLLHKVAG------     11
P07773|CatA|A.baylyi                ----------------------------------MEVKIFNTQDVQDFLRV-AS------     19
O33948|CatA1|A.lwoffii              ----------------------------------MSIKVFGTKEVQDLLKA-AT------     19
Q8GAY6|CatA1|Burkholderia sp.       ----------------------------------MSVKVFDTKEVQDLLKA-AA------     19

A0A060T9I8|Acdo1p|B.raffinosiferm.  ---------EANSKGKDPRMTFIISRLIPHLHDFAREVRLTFEEWQLGLKFLVDVGKTCT     78
A2QAP8|HqdA|A.niger                 ---------RINSLCQDERLKYVLERLVTHLHDFARETRLSTDEWMTGLRFLTEVGKICS     75
A2QEL8|NRRL3_5330|A.niger           ---------KINSMCDNPRMRYLILKLIQAAHDYIRDVGLQFDEWEQAWQFLTRVGQIST     104
A2QNW8|CrcA|A.niger                 ---------NSMGPKTTPRFRQLMTGLIRHVHDFARENELTVDEWMAGVKLLNWAGQMSD     64
Q5B4J8|AN4532|A.nidulans            ---------NAMGENVNPRFRQLMASLIRHVHDFARENELTVDEWMAGVQLMNWAGQMSN     66
Q5AT14|PrcA|A.nidulans              ---------NSMGPKTPERARVILGSLIRHIHDFAREVELTPAEWMLGVEFINSIGKIST     67
A2R1P9|PrcA|A.niger                 ---------NSMGPKTPERARVVLGALIRHIHDFAREVELTSAEWMLGVEFINSIGKIST     67
A0A0B4ZYX0|Cato|C.tropicalis        ---------ASMGPKATPKARRMISSLIQHIHDFARENQLTTEEWLWGVNFINRIGQMSD     61
P86029|Hqd2|C.albicans              ---------TSLGPNATPRAKKLIASLVQHVHDFARENHLTTEDWLWGVDFINRIGQMSD     61
P96984|CatA|R.erythropolis          AATDKFKTARVSCDTSPERAAAIYRDVLSALGEVIHRHEVTYDEYRVLKQWMIDVGE---     73
P95607|CatA|R.opacus                AATDKFKAERATADTSPERLAAIAKDALGALNDVILKHGVTYPEYRVFKQWLIDVGE---     61
Q43984|CatA|A.guillouiae            ---------ATATGEVNLRVQQIVVRLLGDLFQAIEDLNMSQTELWKGLEYLTDAGQ---     65
O33950|CatA2|A.lwoffii              ----------SAINEGNPRTKQIVNRIVRDLFYTIEDLDVQPDEFWTALNYLGDAGR---     62
A7LBQ4|CatA|P.putida                ----------LDHAEGNPRFKQIILRVLQDTARLVEDLEITEDEFWHAIDYLNRLGG---     58
P07773|CatA|A.baylyi                ---------GLEQEGGNPRVKQIIHRVLSDLYKAIEDLNITSDEYWAGVAYLNQLGA---     67
O33948|CatA1|A.lwoffii              ---------NLEGKGGNARSKQIVHRLLSDLFKAIDDLDITPDEVWAGVNYLNKLGQ---     67
Q8GAY6|CatA1|Burkholderia sp.       ---------NMGSEDGSARAKQIVNRLLGDLFKAIDDLDMTPDEIWAGVHYFNKLGQ---     67
                                                      :   :    :           :   :       :   *

A0A060T9I8|Acdo1p|B.raffinosiferm.  DVRHEFILLSDVLGLSVLVDAMSHVKA-K----NATPGTLLGPFHTEDANVFEQGECIVS     133
A2QAP8|HqdA|A.niger                 DVRQEYILLSDILGLSILVDSIDHPKP-P----NSTEGTVLGPFHTHDAEPLTPGASI-S     129
A2QEL8|NRRL3_5330|A.niger           DVRHEFVLLSDILGISALVDALSYPAV-P----GATESSVLGPFHDEEAHSFEYGESITA     159
A2QNW8|CrcA|A.niger                 DKRNEGQLVCDVIGLESLVDEITYTLA-NEAPDAPTATAILGPFFRADTPYRDNGANIVL     123
Q5B4J8|AN4532|A.nidulans            AKRNEGQLVCDVIGLESLVDEITFKLA-DEATDAPTATAILGPFFRADTPFRSNGESIVK     125
Q5AT14|PrcA|A.nidulans              PIRNECHRICDVIGLESLVDEIANRIV-TEQGLSPTSNVILGPFWSPNAPFRELGDSIIQ     126
A2R1P9|PrcA|A.niger                 PIRNECHRICDVIGLESLVDEIANKIV-TEDGVSPTSNVILGPFWSPNAPFRELGDSIIQ     126
A0A0B4ZYX0|Cato|C.tropicalis        DKRNEGILVCDILGLESLVDALTNESE-N---STHTSSAILGPFYLPNSPVYPNGGSIVQ     117
P86029|Hqd2|C.albicans              SRRNEGILVCDIIGLETLVDALTNESE-Q---SNHTSSAILGPFYLPDSPVYPNGGSIVQ     117
P96984|CatA|R.erythropolis          --YGEWPLW-----LDVFVEHQVEDVNYSRNGLAGTKGSIEGPYYVPDAPQLPAV-CTMP     125
P95607|CatA|R.opacus                --GGEWPLF-----LDVFIEHSVEEVLA--RSRKGTMGSIEGPYYIENSPELPSK-CTLP     111
Q43984|CatA|A.guillouiae            --ANELGLLAAGLGLEHYLDLRADEADAKAGITGGTPRTIEGPLYVAGAPESVGF-ARMD     122
O33950|CatA2|A.lwoffii              --SGELGLLAAGLGFEHFLDLRMDEAEAKAGVEGGTPRTIEGPLYVAGAPVSDGH-ARLD     119
A7LBQ4|CatA|P.putida                --RNEAGLLAAGLGIEHFLDLLQDAKDAEAGLGGGTPRTIEGPLYVAGAPLAQGE-ARMD     115
P07773|CatA|A.baylyi                --NQEAGLLSPGLGFDHYLDMRMDAEDAALGIENATPRTIEGPLYVAGAPESVGY-ARMD     124
O33948|CatA1|A.lwoffii              --DGEATLLAAGSGLEKYLDIRLDAADKAEGIEGGTPRTIEGPLYVAGATVHDGV-SKID     124
Q8GAY6|CatA1|Burkholderia sp.       --DGEAALLAAGLGLEKFLDIRMDAEDKAAEITGGTPRTIEGPLYVAGAPVRDGI-SKID     124
                                        *         :.  ::               *   : **     :

A0A060T9I8|Acdo1p|B.raffinosiferm.  KENE---GDPLTLYGTVRD-IHGNPIPNVSIDIWETDETGHYDTQY-D--DRNGPDYRGI     186
A2QAP8|HqdA|A.niger                 HDPA---GEPLLVVCTVKD-THGNPVSDVKIDIWETDSTGHYDVQY-A--GRDGPDGRCI     182
A2QEL8|NRRL3_5330|A.niger           EGTP---GEPTIVRGWIKD-TEGNAVPKALIDVWETDGNGVYDLEY-D--GSEDPNCRGK     212
A2QNW8|CrcA|A.niger                 TPPPD--GEMAFMHGQVVDFATKEPLVGATVEVWQASTNGLYEQQD-P--KQEEFNLRGK     178
Q5B4J8|AN4532|A.nidulans            TAPKDGKGEMAYMAGQVLDFVTKKPLVGAVVEVWQASTNGLYEQQD-P--DQEEFNLRGK     182
Q5AT14|PrcA|A.nidulans              DPNPN--GKVTFMHGVLRDMETGAPIAGAVLDIWQASANGQYDFQD-P--NQSENNLRGK     181
A2R1P9|PrcA|A.niger                 DPNPN--GKVTYMHGVLKDMETGAPIVGAVLDIWQASANGQYDFQD-P--NQSENNLRGK     181
A0A0B4ZYX0|Cato|C.tropicalis        KALPT--DVRCLTSGKVTS-VDGKPLAGAKIEVWQCNSAGFYSQQKEH--DGPDFNLRGT     172
P86029|Hqd2|C.albicans              KAIPT--DVKCFVRGKVTD-TEGKPLGGAQLEVWQCNSAGFYSQQADH--DGPEFNLRGT     172
P96984|CatA|R.erythropolis          MREQDRRSTPLVFSGQVTD-LDGNGLGGATVELWHADEDGFYSQFA-P--NIPEWNLRAT     181
P95607|CatA|R.opacus                MREEDEKITPLVFSGQVTD-LDGNGLAGAKVELWHADNDGYYSQFA-P--HLPEWNLRGT     167
Q43984|CatA|A.guillouiae            DGSESAHVDALIIEGNVTD-TAGQIIPNAKVEIWHANSLGNYSFFD-K--SQSAFNLRRS     178
O33950|CatA2|A.lwoffii              DGTDPGQ--TLVMRGRVFG-EDGKPLANALVEVWHANHLGNYSYFD-K--SQPAFNLRRS     173
A7LBQ4|CatA|P.putida                DGTDPGV--VMFLQGQVFD-ADGKPLAGATVDLWHANTQGTYSYFD-S--TQSEYNLRRR     169
P07773|CatA|A.baylyi                DGSDPNG-HTLILHGTIFD-ADGKPLPNAKVEIWHANTKGFYSHFD-PTGEQQAFNMRRS     181
O33948|CatA1|A.lwoffii              INPDEDA-GPLVIHGTVTG-PDGKPVAGAVVECWHANSKGFYSHFD-PTGAQSDFNLRGA     181
Q8GAY6|CatA1|Burkholderia sp.       VNPDEGA-GPLVIRGTVTG-PDGKPVANALVECWHANSKGFYSHFD-PTGAQSEFNLRGA     181
                                                    : .      :  . :: *. .  * *.            : *

A0A060T9I8|Acdo1p|B.raffinosiferm.  IYTDAEGKYLIKGIVPVSYPIPHDGPVGRFLTYVGRHPYRPAHIHFKLEKEGYDNLITGL     246
A2QAP8|HqdA|A.niger                 MTSDKEGVFWFKAITPVPYPIPHDGPVGKLLKLLGRHPYRPSHMHFMFEKGGFDHLITAL     242
A2QEL8|NRRL3_5330|A.niger           IFSDERGHYLFSCVKPVAYPISNDGPVGELLRKLKRHWFRPAHMHFMVAHPEYTKLITAL     272
A2QNW8|CrcA|A.niger                 FKTDEQGRYSFYCLRPTPYPVPDDGPAGKLLELMDRHPFRPAHIHIIATYDGYKPLTTQI     238
Q5B4J8|AN4532|A.nidulans            FRTDENGRYSFYCLRPTPYPVPDDGPAGKLLKLMDRHPFRPAHIHIIATHDGYKPLTTQI     242
Q5AT14|PrcA|A.nidulans              FRSNEKGEFYWYCYHPTPYSLPTDGPAGVLLNLMDRSPMRPAHIHLMITHPDYATVINQI     241
A2R1P9|PrcA|A.niger                 FRSNEKGEFNWYCYHPTPYSLPTDGPAGVLLNLMDRSPMRPAHIHLMITHPDYATVINQI     241
A0A0B4ZYX0|Cato|C.tropicalis        FYTDDDGNYSFECLKPTSYPIPYDGPAGDLLLLMDRHPHRPSHIHWRVSHPEYHTLVTQI     232
P86029|Hqd2|C.albicans              FITDDEGNYSFECLRPTSYPIPYDGPAGDLLKIMDRHPNRPSHIHWRVSHPGYHTLITQI     232
P96984|CatA|R.erythropolis          IVCDDEGRYEITTIQPAPYQIPHDGPTGWFIESHGGHPWRPAHLHLMVKAPGRLPITTQL     241
P95607|CatA|R.opacus                IIADEEGRYEITTIQPAPYQIPTDGPTGQFIEAQNGHPWRPAHLHLIVSAPGKESVTTQL     227
Q43984|CatA|A.guillouiae            IFTDTQGQYIAQTTMPVGYGCPPEGTTQALLNLLGRHGNRPSHVHYFVSAPGYRKLTTQF     238
O33950|CatA2|A.lwoffii              IRTDAEGKYSFRSVVPVGYSVPPQGQTQLLLDQLGRHGHRPAHIHFFVSAPGFRKLTTQI     233
A7LBQ4|CatA|P.putida                IITDAEGRYRARSIVPSGYGCEPAGPTQECLDLLGRHGQRPAHVHFFISAPGFRHLTTQI     229
P07773|CatA|A.baylyi                IITDENGQYRVRTILPAGYGCPPEGPTQQLLNQLGRHGNRPAHIHYFVSADGHRKLTTQI     241
O33948|CatA1|A.lwoffii              VKTGADGKYEFRTLMPVGYGCPPQGATQQLLNVLGRHGNRPAHVHFFVSSDSARKLTTQF     241
Q8GAY6|CatA1|Burkholderia sp.       VSTDVDGKYEFRTLMPVGYGCPPHGATQQLLNVLARHGNRPAHVHFFVTTDKYRKLTTQI     241
                                    .  .  * :      *  *     * .   :        **:*:*          : . :

A0A060T9I8|Acdo1p|B.raffinosiferm.  YMKGDKYSGEDAVFGEKKELTVEPKKFGDQKLA-EKYMS-----------NADDYFINFD     294
A2QAP8|HqdA|A.niger                 YLRNDPYETSDAVFGVKDSLVVDIGKAGPE-YA-AKYGV-----------SEDHALLTYD     289
A2QEL8|NRRL3_5330|A.niger           YSRDSNFVESDTVFGVKKSLIVDYNWCEDLELA-KQHNVEPIVKTIDGRESKGFWLLERD     331
A2QNW8|CrcA|A.niger                 FDSKDKYLTNDSVFAVKDSLIVDFVPRKDDPQ--------------------AGLELNYD     278
Q5B4J8|AN4532|A.nidulans            FDRKDPYLTNDSVFAVKDSLIVDFVERKGDPQ--------------------AGLELKYD     282
Q5AT14|PrcA|A.nidulans              YPSDDPHLDIDSVFAVKDDLVVDFKPKTDDPK--------------------AELDLEYN     281
A2R1P9|PrcA|A.niger                 YPSDDPHLDIDSVFAVKDDLVVDFKPKTDDPK--------------------AQLDLEYN     281
A0A0B4ZYX0|Cato|C.tropicalis        YDSECPYTKNDSVHAVKDDIIVEFKERDG------------------------GWYLDYD     268
P86029|Hqd2|C.albicans              YDAECPYTNNDSVYAVKDDIIVHFEKVDNKDK----DLV-----------GKVEYKLDYD     277
P96984|CatA|R.erythropolis          YFRGGDWVETDVATAVKPELVLDPVRGADGVN-----------------------RVAYD     278
P95607|CatA|R.opacus                YFKGGEWIDSDVASATKPELILDPKTGDDGKN-----------------------YVTYN     264
Q43984|CatA|A.guillouiae            NIEGDKYLWDDFAFATRDGLIATALDVTDLAK-IKQYNL-----------NKAFKHIKFN     286
O33950|CatA2|A.lwoffii              NIDGDPYLWDDFAFATRDGLVPAVRQAEVRKANRTAWTV-----------SSR-------     275
A7LBQ4|CatA|P.putida                NFEGDKYLWDDFAYATRDGLIGELRFVEDAAAARDRGVQ-----------GERFAELAFD     278
P07773|CatA|A.baylyi                NVAGDPYTYDDFAYATREGLVVDAVEHTDPEA-IKANDV-----------EGPFAEMVFD     289
O33948|CatA1|A.lwoffii              NIEGDPLIWDDFAYATREELIPPVTEKKGGTA-LGL--K-----------ADTYKDIEFN     287
Q8GAY6|CatA1|Burkholderia sp.       NIEGDPLIWDDFAYATREDLIPHVVEKTGGTP-LGM--K-----------ADTYKEIEFN     287
                                              * . . :  :

A0A060T9I8|Acdo1p|B.raffinosiferm.  ITLMSTKESMELFDAQSRRDLKTLGIDAKVVNGVPIADLD             334
A2QAP8|HqdA|A.niger                 FVLVSDEETSELRARNSKEALDKLGRKVKIVNGLPVPDLD             329
A2QEL8|NRRL3_5330|A.niger           FILVKKSPPPPR----KTEDLE------------------             349
A2QNW8|CrcA|A.niger                 VKLVRAPAN-------------------------------             287
Q5B4J8|AN4532|A.nidulans            VKLVPDGMKSNGA---------------------------             295
Q5AT14|PrcA|A.nidulans              VKMALKKHHPNPNSAPPVSSFERYNKAGKEKL--------             313
A2R1P9|PrcA|A.niger                 VTMALKKHHPNPNSAPPVSSFERFNKASKTQEKL------             315
A0A0B4ZYX0|Cato|C.tropicalis        ISLATEESIKRARENKK-----------------------             285
P86029|Hqd2|C.albicans              ISLATESSIQEARAAAKAR--------QDAEIKL------             303
P96984|CatA|R.erythropolis          FALDPTP---------------------------------             285
P95607|CatA|R.opacus                FVLDPA----------------------------------             270
Q43984|CatA|A.guillouiae            FQLVQDADQVPLQRLIV-------------VE--------             305
O33950|CatA2|A.lwoffii              ----------------------------------------             275
A7LBQ4|CatA|P.putida                FHLQAAAAPEAEARSHRPRA---------LQEG-------             302
P07773|CatA|A.baylyi                LKLTRLVDGVDNQVVDRPRL---------AV---------             311
O33948|CatA1|A.lwoffii              LTLTSLVKGKDNQVVHRLRA---------EVAA-------             311
Q8GAY6|CatA1|Burkholderia sp.       IELTPLVHGKDNQLVSRLRA---------SVTA-------             311
```

Supplementary Figure 10 Sequence alignment of bacterial and fungal intradiol ring cleavage dioxygenases. (See Figure 1 for details.)
